# The impact of narcolepsy and idiopathic hypersomnia on work-related outcomes: a systematic literature review

**DOI:** 10.1093/sleepadvances/zpag078

**Published:** 2026-07-17

**Authors:** Somraj Ghosh, Stephen Crawford, Sarah L Bermingham, Miriam L Zichlin, Fiona Stewart, Nicole Fusco, Evelyn Gomez-Espinosa, Kristin Kistler

**Affiliations:** Takeda Development Center Americas, Inc, Cambridge, MA, United States; Takeda Development Center Americas, Inc, Cambridge, MA, United States; Takeda Pharmaceuticals USA, Inc, Cambridge, MA, United States; Takeda Development Center Americas, Inc, Cambridge, MA, United States; Cencora Global Consulting Services, Conshohocken, PA, United States; Cencora Global Consulting Services, Conshohocken, PA, United States; Cencora Global Consulting Services, Conshohocken, PA, United States; Cencora Global Consulting Services, Conshohocken, PA, United States

**Keywords:** work productivity, narcolepsy, idiopathic hypersomnia, work-related outcomes, work satisfaction

## Abstract

The symptoms experienced by people with narcolepsy and idiopathic hypersomnia (IH) have profound effects on individuals’ lives, affecting daily functioning, relationships and mental health, and can impair performance at work or school. Although numerous studies have evaluated the impact of narcolepsy and IH on work-related outcomes, the evidence base is highly fragmented and difficult to meaningfully summarize. We conducted a systematic literature review of published evidence to better understand the impact of these disorders on employment, work productivity, work satisfaction, and related outcomes. We identified 54 publications representing 51 unique studies that met the study inclusion criteria and were included in the review. Although most people with narcolepsy or IH were employed, fewer were in paid work than control populations, and absenteeism and presenteeism were high. Having narcolepsy and IH resulted in lower wages and missed work time, leading to unfavorable impacts on career progression, promotion, job satisfaction, and income. The most frequently reported work-related problems were sleepiness at work, reduced performance, work accidents, and lateness. More severe excessive daytime sleepiness was associated with greater work impairment. Narcolepsy-specific medications that improved excessive daytime sleepiness were reported to improve work productivity in clinical trials; however, many people are left with substantial residual impairment despite treatment. These findings indicate that narcolepsy and IH impose a substantial burden on work functioning and employment, and there is a need for more research to inform interventions and policies aimed at mitigating the impact of these debilitating conditions on individuals’ professional lives.

## Graphical Abstract

**Graphical Abstract:**
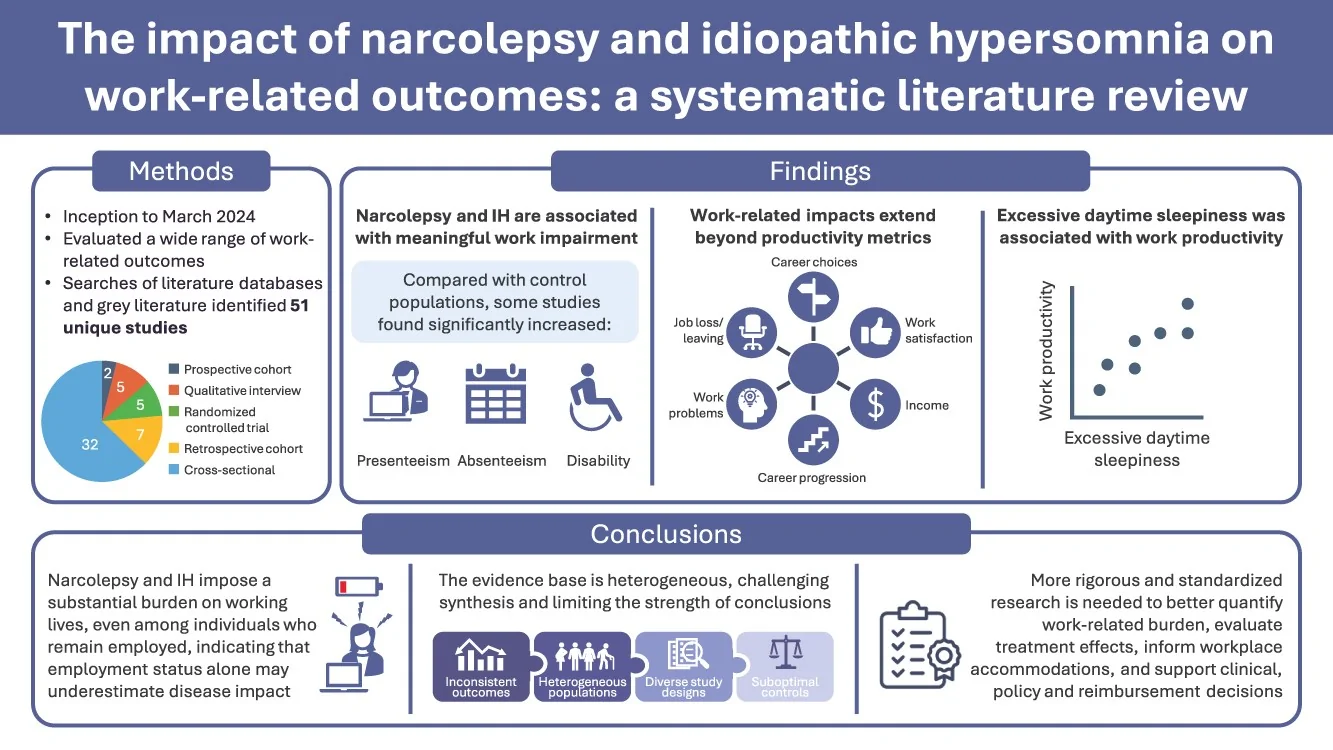

## 1. Introduction

Narcolepsy and idiopathic hypersomnia (IH) are chronic sleep disorders classified as central disorders of hypersomnolence, with excessive daytime sleepiness (EDS) as a core diagnostic criterion [1]. Narcolepsy is subdivided into narcolepsy type 1 (NT1), characterized by cataplexy and orexin deficiency, and narcolepsy type 2 (NT2), which lacks cataplexy and normal-intermediate orexin levels. Both narcolepsy subtypes frequently involve disrupted nighttime sleep, hypnagogic hallucinations and sleep paralysis, with refreshing naps [2]. In contrast, IH is marked by prolonged nocturnal sleep durations, severe sleep inertia, unrefreshing naps, and has an unknown etiology [3, 4]. Autonomic dysfunction and cognitive symptoms are common across all 3 conditions [3, 5, 6]. Narcolepsy and IH profoundly impact the health, daily life, work, education, relationships, and well-being of those who experience these conditions [4, 6–8].

Symptoms of narcolepsy and IH typically emerge between late adolescence and early adulthood, which is a critical developmental period when individuals are making career decisions and establishing their professional paths [6, 9]. The mean time from symptom onset to diagnosis averages from 8 to 15 years [10, 11], leaving individuals with NT1, NT2, and IH to manage debilitating symptoms without support [10, 12]. Even after diagnosis, current treatments only partially reduce a limited scope of symptoms [13]. As a result, many individuals with these conditions encounter lifelong challenges related to workforce participation, productivity, and career impact [14, 15].

Numerous studies have examined the impact of narcolepsy and IH on work-related outcomes [15–19]. Most people living with these conditions report that it interferes with work [8, 20]. However, evidence of the extent and causes of these challenges remains fragmented and difficult to interpret. Heterogeneity in study designs, populations, and outcome definitions further contribute to conflicting findings and limit comparability between studies. There is a need to systematically identify and organize the existing evidence, assess its quality, summarize the data, and identify key areas of uncertainty in order to provide a clearer understanding of how these conditions affect individuals’ working lives, help inform workplace accommodations, optimize treatment strategies, and guide policy decisions.

To address these gaps, we conducted a systematic review of the literature to better understand the impact of narcolepsy and IH on outcomes such as workforce participation, work productivity, and career impact.

## 2. Objectives

This review aimed to systematically identify and synthesize evidence on the impact of NT1, NT2, and IH on a broad range of work-related outcomes. Specifically, the review addressed (1) the prevalence of work-related impairments across a comprehensive set of predefined outcomes selected from scoping searches of the current literature; (2) differences in work-related outcomes between affected individuals and healthy controls; (3) factors contributing to work-related challenges as reported by people living with these conditions; and (4) associations between symptom severity, health-related quality of life (HRQoL), and treatment and work-related outcomes.

## 3. Methods

This review was conducted in accordance with the Cochrane Handbook for Systematic Reviews and reported following the Preferred Reporting Items for Systematic reviews and Meta-Analyses (PRISMA) guidelines [21, 22].

### 3.1. Eligibility criteria

Eligibility criteria were informed by a preliminary scoping review and expert input using the Population, Intervention, Comparator, Outcomes, Study design (PICOS) framework (Table S1). Studies were included if they involved humans of any age diagnosed with narcolepsy (NT1 or NT2) or IH, with no restrictions on interventions or comparators. A wide range of work-related outcomes were specified, including absenteeism, presenteeism, job loss, income, career progression, work satisfaction, as well as relationships between these outcomes and symptom severity, HRQoL, and treatment. Eligible study designs included observational, interventional, and qualitative research. Any study with fewer than 10 participants and any studies explicitly described as case studies were excluded.

### 3.2. Information sources

Systematic literature searches were conducted in Ovid to identify studies of interest in the following databases: Embase, Emcare, Econlit, Medline, Cochrane Central Register of Controlled Studies, and Cochrane Database of Systematic Reviews. Grey literature and citations in previous systematic literature reviews were also reviewed non-systematically. Databases were searched from inception to March 31, 2024. No language limits were applied.

### 3.3. Search strategy

Search strategies were developed and iteratively refined using a combination of controlled vocabulary (MeSH and Emtree terms adapted for each database) and free-text terms covering the populations and outcomes of interest. Free-text terms were identified from synonyms, existing Cochrane reviews, previously identified relevant studies from the scoping search, and background literature. Draft strategies were piloted to ensure retrieval of key references. Validation included checking citation lists of relevant review articles, which did not identify additional eligible studies. Search strings for each database are provided in Table S2. Reference lists of relevant review articles were also checked for additional publications meeting inclusion criteria.

### 3.4. Screening, data collection and data extraction

All titles and abstracts retrieved by the literature searches were screened by 2 independent researchers, with conflicts resolved by a third independent researcher. Records deemed potentially relevant were retrieved in full-text and screened for eligibility by 2 independent researchers, with conflicts resolved by a third independent researcher. Data on selected outcomes (Table S1) from the included studies were extracted into a Microsoft Excel spreadsheet by one researcher, followed by validation of all extracted data by a second researcher. For multiple papers reporting data from the same studies, each paper reported distinct outcomes, with no inconsistencies identified. Data were evaluated by population: people with unspecified narcolepsy type (PwUN), people with NT1 (PwNT1), people with NT2 (PwNT2), and people with IH (PwIH).

### 3.5. Evidence synthesis

Evidence synthesis was conducted using a narrative approach, structured around patient populations (PwUN, PwNT1, PwNT2, and PwIH). It was not possible to conduct a meta-analysis due to the heterogeneity of the study designs, populations, interventions and outcomes, but median estimates and/or ranges were determined where appropriate.

### 3.6. Critical appraisal

Each included study was critically appraised using a validated tool appropriate to its design. One researcher conducted the assessment manually, and all ratings were independently reviewed and validated by a second researcher. The tools used were the Joanna Briggs Institute Critical Appraisal Checklist for Analytical Cross-Sectional Studies (including qualitative studies) [23], International Society for Pharmacoeconomics and Outcomes Research Task Force Checklist for retrospective cohort studies [24], Newcastle-Ottawa Scale for prospective cohort studies [25], and Cochrane risk-of-bias tool for RCTs [26].

## 4. Results

### 4.1. Search results

**Figure 1:**
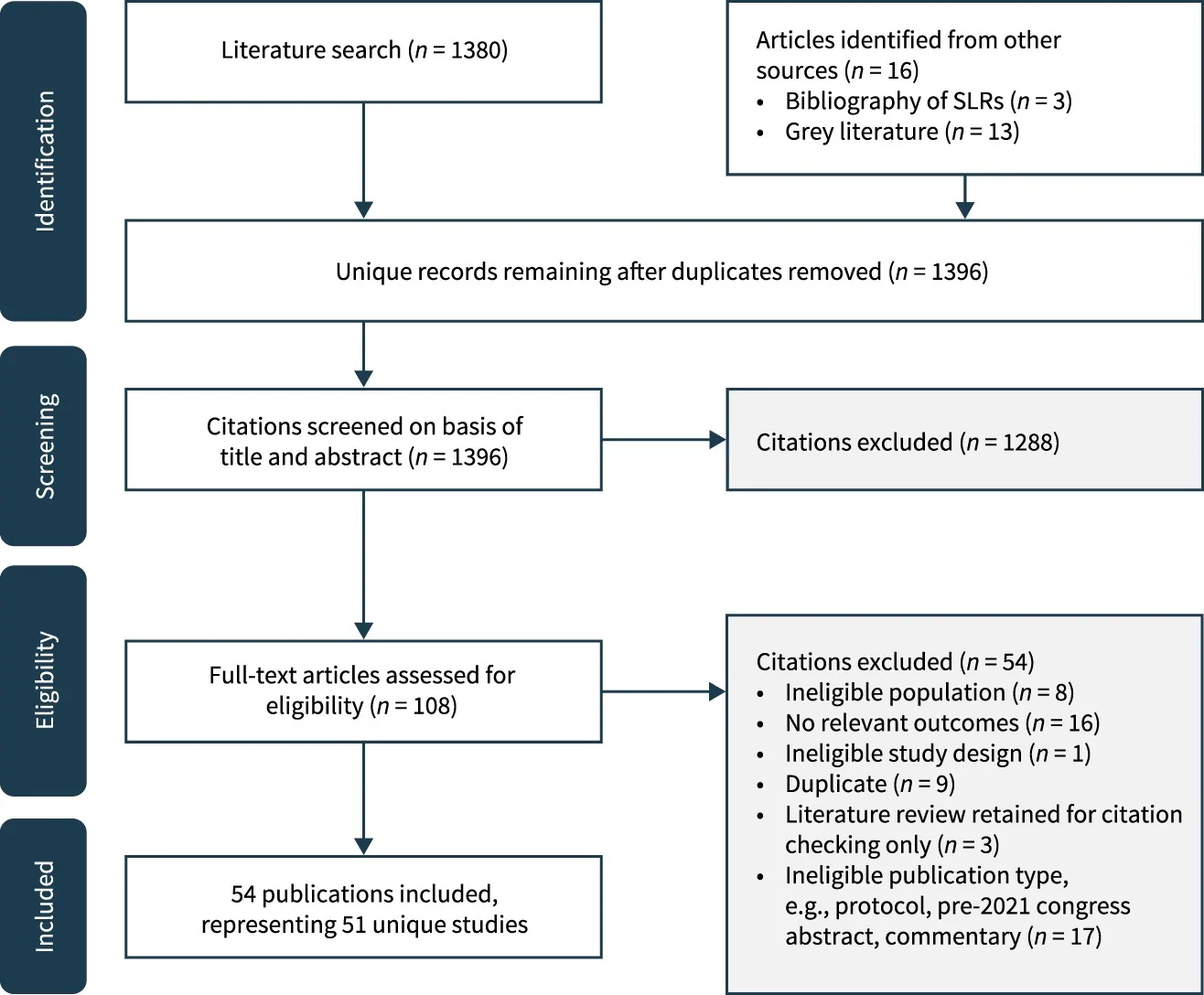
PRISMA diagram. SLR, systematic literature review.

Database searches yielded a total of 1380 records, and 16 articles were identified from other sources (grey literature and citations in previous systematic literature reviews), resulting in a total of 1396 records being screened. Of these, 108 were included for full-text screening, and 54 publications representing 51 unique studies met the study inclusion criteria and were included in the review (Figure 1).

### 4.2. Study characteristics

#### 4.2.1. Description of studies

##### 4.2.1.1. Study design

The identified studies represented a broad range of study designs. Of 51 studies, 32 were cross-sectional (predominantly survey and questionnaire based) [8, 15, 16, 18, 27–57], 7 were retrospective cohort studies [58–64], 5 were randomized clinical trials (RCTs) [17, 19, 65–67], 5 were qualitative interview studies [68–72], and 2 were prospective cohort studies [73, 74] (Table 1). Of the 19 studies that included a control arm, 17 included healthy control populations; 14 were age- and sex-matched; 6 comprised relatives, friends or neighbors; and 2 included control individuals with other disorders (epilepsy and obstructive sleep apnea/hypopnea syndrome). Most studies were conducted in the United States (14 studies) or Europe (22 studies) (Table 2). A summary of the included studies is provided in Table 2, with detailed characteristics in Table S3.

**Table 1:** Study and participant characteristics

	Studies (*N* = 51)
Study type	
Cross-sectional studies [8, 15, 16, 18, 27–57]	32^a^
Retrospective cohort studies [58–64]	7
Randomized controlled trials [17, 19, 65–67]	5
Qualitative interview studies [68–72]	5
Prospective cohort studies [73, 74]	2
Average age across all studies, years	
Mean (range)	41 (21–72)
Female, median (range), %	
Narcolepsy	55 (19–93)
IH	68 (22–81)
Sample sizes, *n* range	
Narcolepsy	15–9312
IH	20–2855

^a^From 35 publications.

*Abbreviation:* IH, idiopathic hypersomnia.

**Table 2:** Summary of included studies

Author, year	Study design	Country	Sample size
Black 2014 [59]	RC	United States	Overall: 9312 With productivity data: 600
Bae 2019 [73]	PC	United States	102
Ferrans 1992 [38]	CS	United States	539
Flores 2016 [39]	CS	United States	437
Goswami 1998 [41]	CS	United States	68
Jones 2016 [68] (dissertation)	Qual CS	United States	15
Kapella 2015 [45]	CS	United States	122
Maski 2017 [8]	CS	United States	NT1: 997 NT2: 329 PwUN: 149 Total: 1697
Rogers 1984 [70]	Qual CS	United States	NT1: 16 NT2: 4 Total: 20
Ton 2014 [55]	CS	United States	NT1: 152 NT2: 74 Total: 226
Ong 2021 [69]	CS	United States	NT1: 17 NT2: 12 Total: 29
Rodrigues Aguilar 2021 [52]	CS	Brazil	NT1: 52 NT2: 24 Total: 76
Rovere 2008 [53]	CS	Brazil	40
Broughton 1984 [31]	CS	Canada	60
Jennum 2009 [63]	RC	Denmark	462
Jennum 2012 [61]	RC	Denmark	816
Jennum 2020 [74]	PC	Denmark	171
White 2020 [57]	CS	France	NT1: 51 NT2: 18 Total: 69
Kallweit 2022 [64]	RC	Germany	413
Müller 2021 [47]	CS	Germany	133
Dodel 2004 [36] and Dodel 2007 [35]	CS	Germany	75
Geremew 2017 [40]	CS	Iran	NT1: 19 NT2: 25 Total: 44
Campbell 2011 [32]	CS	New Zealand	54
David 2012 [34]	CS	Portugal	51
Abenza 2023 [58]	RC	Spain	NT1: 27 NT2: 27 Total: 54
Daniels 2001 [33]	CS	United Kingdom	305
Teixeira 2004 [54]	CS	United Kingdom	49
Broughton 1981 [28] and Broughton 1983 [29]	CS	Canada, Czech Republic, Japan	180
Emsellem 2020 [19]	RCT	United States, Canada, Finland, France, Germany, Italy	177
Ohayon 2016 [48] and Thorpy 2021 [16]	CS	International (92% United States)	Initial report: 546 Final report: NT1: 661 NT2: 322 Total: 983
Weaver 2021 [66]	RCT	Europe; North America	231
Weaver 2021 [65]	OLE of RCT	Europe, North America	278
Alaia 1992 [27]	CS	United States	102
Kales 1982 [44]	CS	United States	50
Kovalská 2016 [46]	CS	Czech Republic	42
Peter-Derex 2024^*^ [51, 75]	CS	France	235
Ingravallo 2012 [42]	CS	Italy	100
Ingravallo 2008 [43]	CS	Italy	15
Bassi 2023 [15]	CS	Italy	127
Overeem 2011 [49]	CS	Netherlands	116
Ervik 2006 [37]	CS	Norway	77
Stevens 2023 [18]	CS	United States	75
Broughton 1978 [30]	CS	Czech Republic	30
Jennum 2010 [62]	RC	Denmark	2208
Jennum 2014 [60]	RC	Denmark	2855
Morse 2023 [17]	OLE of RCT	Belgium, Czech Republic, Finland, France, Poland, Spain, United States	154
Thorpy 2022 [67]	RCT	Europe; North America	154
Srivastava 2021 [71]	Qual	NR	20
Susta 2022 [72]	CS	NR	43
Ozaki 2012 [50]	CS	Japan	NT1: 83 NT2: 48 IH: 54 Total: 185
Wasling 2020 [56]	CS	Sweden	NT1: 68 IH: 21 Total: 89

*Abbreviations:* CS, cross-sectional study; NR, not reported; NT1, narcolepsy type 1; NT2, narcolepsy type 2; OLE, open-label extension; PC, prospective cohort study; PwUN, people with unspecified narcolepsy type; Qual, qualitative; RC, retrospective cohort study; RCT, randomized controlled trial.

^*^Available as a non-peer-reviewed preprint at the time of the literature search [51]. The article was subsequently published in a peer-reviewed journal, and the citation has been updated in this review [75].

##### 4.2.1.2. Study populations

Overall, 44 studies included participants with any narcolepsy, and 10 studies included participants with IH (3 included participants with both narcolepsy and IH). Four studies reported data separately for participants with NT1 and NT2, 9 for NT1 only, 7 for IH only, and 2 reported data separately for NT1, NT2, and IH. The type of narcolepsy (NT1 or NT2) was not specified in 30 studies. Sample sizes of studies involving people with narcolepsy ranged from 15 [68] to 9312 [59] (median, 102 [44 studies]). For studies involving PwIH, sample sizes ranged from 20 [71] to 2855 participants [60] (median, 106 [10 studies]).

##### 4.2.1.3. Data collection

Data collection periods spanned several decades, from the 1970s through the 2020s. Among studies reporting collection periods, the earliest began in 1986 [41] and the most recent ran from 2020 to 2023 [75]. Several early publications did not report data collection dates.

### 4.3. Participant characteristics

#### 4.3.1. Age and sex

Substantial variation was observed across study populations. The proportion of females ranged from 19% to 93% (median, 55%) in people with narcolepsy, and from 22% to 81% (median, 68%) in PwIH (Table 1). The average age of participants, calculated at the study level, ranged from 27 years in a study of young adults [45] to 72 years in a study of individuals older than 60 years [46]. The median age across all study-level averages was 41 years.

#### 4.3.2. Diagnostic criteria

Criteria for defining narcolepsy and IH cases varied between studies. Narcolepsy cases were defined using International Classification of Sleep Disorders (ICSD) criteria in 19 studies (16 cross-sectional studies and 3 RCTs): 5 used ICSD, Third Edition, criteria [15, 19, 56, 57, 75]; 7 used ICSD, Second Edition, criteria [34, 40, 42, 43, 46, 49, 50]; 3 specified that either ICSD, Second or Third Edition, criteria could be used [17, 67, 72]; and 4 used the original ICSD [36, 37, 53, 54]. In 2 claims-based studies, narcolepsy cases were identified using *International Classification of Diseases* (*ICD*), *Tenth Revision* codes [47, 64] and 1 used *ICD-9* codes [59]. Three studies reported using different criteria: 1 used the American Academy of Sleep Medicine guidelines [52], 1 used Roth criteria for diagnosing IH [30], and 1 used the presence of “both excessive daytime sleepiness or sleep attacks and cataplexy symptoms” [27]. Criteria for confirming narcolepsy or IH were not reported for the remaining studies [8, 18, 27, 28, 30–33, 38, 39, 41, 44, 45, 55, 58, 60–63, 68–74].

#### 4.3.3. Comorbidities

The proportion of individuals with depression was reported in 16 studies and ranged from 12% to 67% (median, 53%). Regarding severity of depression, 2 studies reported mean Beck Depression Inventory scores of 11.1 [15] and 13.1 [75] (score >9 indicates mild depression) and another reported a mean score of 32.9 on the Zung Self-Rating Scale, indicative of normal/non-depressed [43] (≥50 indicates mild depression). One study stipulated elevated symptoms of depression, defined as a total score of 10 or more on the Patient Health Questionnaire-9, as an inclusion criterion [69].

#### 4.3.4. Medications

Across 21 studies (23 publications) reporting prior or current non-investigational treatments, most individuals were receiving pharmacotherapy for EDS and/or cataplexy (range, 41–94%; median across studies, 87%) [8, 15, 16, 18, 27–30, 32, 33, 37, 42, 43, 46–49, 52, 54, 56, 57, 70, 75]. Stimulants were the most commonly used class (range, 30–91% from 15 studies) [15, 16, 30, 32, 33, 37, 42, 43, 46, 52, 54, 56, 57, 70, 75]. Use of specific agents ranged as follows: oxybates, from 4% to 100% (10 studies) [8, 15, 17, 42, 46, 47, 56, 57, 73, 75]; modafinil, from 19% to 87% (6 studies) [8, 43, 47, 56, 57, 75]; and pitolisant, from 19% and 21% (2 studies) [57, 75]. Conversely, the proportion receiving no pharmacotherapy ranged from 8% to 59% individuals (11 studies [12 publications]) [15, 16, 27–30, 33, 37, 43, 47, 52, 70].

### 4.4. Methodological quality assessment

Methodological quality assessment was performed on all studies; however, 5 post hoc clinical RCT analyses (e.g., open-label extension studies or pooled analyses) were exploratory in nature and were not planned *a priori* for standard assessment; thus, quality assessment could not be completed. Methodological quality assessment was also not performed for one abstract and one dissertation. Amongst the 32 cross-sectional studies, definitions of inclusion criteria, descriptions of study participants and settings, and methods of outcome measurements were generally high quality, although there were concerns related to a lack of adjustments for confounding factors and 3 studies did not use reliable or valid methods to measure the condition (i.e., self-reported diagnoses). In the 7 retrospective cohort studies, there were concerns about the appropriateness of the statistical methods used, the lack of reporting of linkage processes in the claims databases and concerns due to a lack of clarity around the link between the natural history of the disease and data collection periods for the studies. There were few concerns in these studies about generalizability, sample selection, and data source reliability and validity. The overall risk of bias in the one prospective cohort study was low. The single RCT had some concerns about randomization and allocation concealment, due to a lack of reporting and unexpected imbalances in attrition between groups. The 3 qualitative cross-sectional studies were judged to have a high risk of bias and limited generalizability, owing in part to a lack of reported measures; however, the representation of patient voices was considered appropriate. Full results are presented in Figure S1.

### 4.5. Workforce participation

#### 4.5.1. Employment

**Figure 2:**
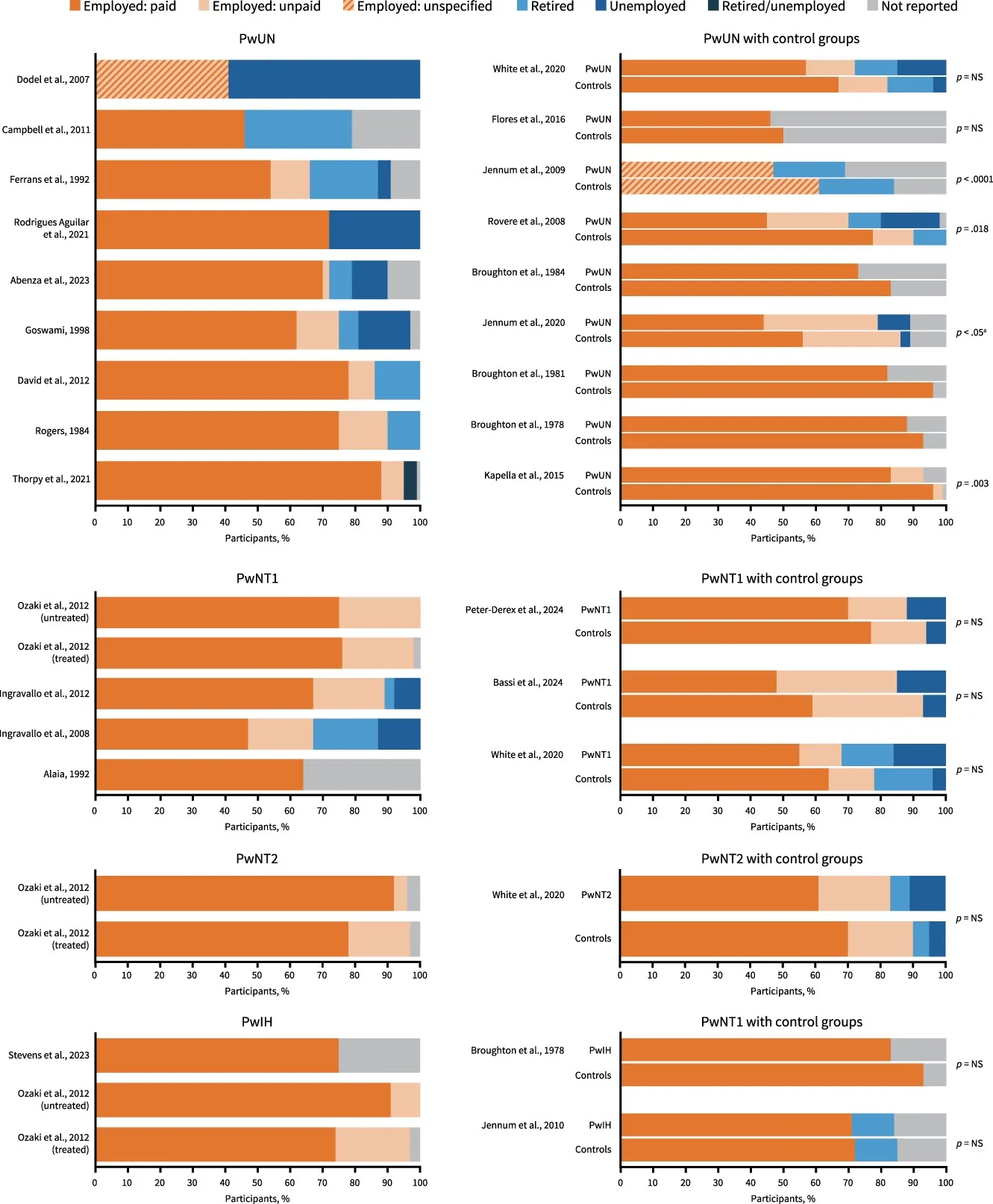
Employment status. ^a^*p*-value PwUN versus placebo for proportion of unemployed individuals only. NS, non-significant; PwIH, people with idiopathic hypersomnia; PwNT1, people with narcolepsy type 1; PwNT2, people with narcolepsy type 2; PwUN, people with unspecified narcolepsy type.

The proportion of people with narcolepsy and IH considered to be employed during the study periods varied considerably, depending on the definition of “employment” utilized (Figure 2), i.e., whether students, homemakers, retirees, and those receiving disability support were considered separately, and whether or not these groups were classified as employed. Among PwUN, 41–96% were considered to be employed, whether unpaid (e.g., students and homemakers) or in paid work across 17 studies [16, 28, 30–32, 34, 35, 38, 39, 41, 45, 52, 53, 58, 63, 70, 74]. Of these, 44–88% were specifically reported to be in paid work across 15 studies [16, 28, 30–32, 34, 38, 39, 41, 45, 52, 53, 58, 70, 74]. For PwNT1, 64–100% were considered to be in any employment, with 47–76% of PwNT1 specifically reported to be in paid work across 7 studies [15, 27, 42, 43, 50, 57, 75]. Two studies reported that 83–96% of PwNT2 were in any employment, and 61–92% were reported to be in paid work [50, 57]. Among PwIH, 71–100% were in any employment and 71–91% were in paid employment across 4 studies [18, 30, 50, 62].

Of 18 studies reporting employment data in PwUN, 9 included controls, who came from a variety of groups including patients, family, friends, hospital workers, and other volunteers [28, 30, 31, 39, 45, 53, 57, 63, 74]. All 9 studies with controls reported lower employment in PwUN compared with controls, with 4 showing statistically significant differences [45, 53, 63, 74] and 5 not reaching statistical significance [28, 30, 31, 39, 57] (Figure 2). Among studies in PwNT1, 3 included comparator groups drawn from friends, relatives, or acquaintances [15, 57, 75]; none found significant differences in employment rates. Two studies found no significant differences in paid employment in PwIH (71–83%) versus healthy controls (72–93%) [30] and 1 study reported a small but non-significant decrease in the proportion of PwNT2 in paid employment versus healthy controls (61% vs. 70%) [57].

Where reported, most employed people with narcolepsy and IH worked full-time. Among PwUN, one study reported an average of 40 h per week at a primary job and 3 h at additional jobs [32], and another reported that 42% worked at least 35 h per week [45]. In PwNT1, full-time employment ranged from 45% to 81% [15, 50], and the mean number of hours of work per week ranged from 26 to 40 h [15, 42, 56].

#### 4.5.2. Unemployment

As with employment, definitions of unemployment varied between studies with respect to the inclusion of students, homemakers, retirees, and those receiving disability benefits.

A minority of people with narcolepsy and IH were unemployed (Figure 2). Among PwUN, 4–59% overall were reported to be unemployed (including retired and disabled) across 9 studies [32, 35, 38, 41, 52, 53, 58, 70, 74]; 4–28% were specifically reported as unemployed (not including retired and disabled) across 6 studies [38, 41, 52, 53, 58, 74], and 7% to 33% were specifically reported as being retired across 8 studies [32, 34, 38, 41, 53, 58, 63, 70]. Two studies with control groups reported significantly higher unemployment in PwUN compared with controls (10% vs. 3% [74] and 18% vs. 0% [53]).

In PwNT1, unemployment ranged from 8% to 16% across 5 studies [15, 42, 43, 57, 75]. The proportion of PwNT1 reported as being retired ranged from 3% to 20% across 3 studies [42, 43, 57]. Three studies reported higher unemployment for PwNT1 compared with control groups selected from parents, children, partners, friends, or acquaintances of study participants (15% vs. 7%, *p =* 0.03 [15]; 12% vs. 6%, (p value not reported) [75]; and 16% vs. 4%, *p-*value non-significant [57]), and 1 study compared unemployment in PwNT1 with the general Italian population (8% vs. 5%) but did not assess statistical significance [42].

Among PwNT2, one study found that 11% were unemployed versus 5% of controls, a non-significant difference [57]. No studies in PwIH reported unemployment rates.

#### 4.5.3. Early retirement

Evidence on early retirement is limited. For PwUN and PwIH, similar proportions were reported to receive early pension benefits as controls [62, 63]. In another study, no PwNT1 reported early retirement [42]. No data on early retirement were reported for PwNT2.

#### 4.5.4. Disability

Nine studies reported disability among PwUN; 34% had obtained official recognition of disability in 1 study [57], 7–14% were described as disabled (self-reported in 2 studies [38, 39], and method of reporting unspecified in 1 study [58]), and 8–16% were receiving disability payments [28, 30, 31, 45, 57, 74]. Of these 9 studies, 7 included control groups, of which 6 reported a higher proportion of PwUN with disability versus controls, although only 1 reported a statistically significant difference [39] (Table 3). Among PwNT1, 31–41% had an official recognition of disability [15] and 15% considered themselves permanently disabled [27]. Regarding disability payments, 10–13% were receiving a disability pension [46], 38% were receiving sickness/disability compensation [56], 47% had applied for social benefits [43], and 54% of retirees received a disability pension before retirement [46]. Of the 6 studies reporting the proportion of PwNT1 with disability, 3 included control groups [15, 46, 75], all of which reported higher rates of disability among PwNT1, with 2 reaching statistical significance [15, 75] (Table 3). No studies reported disability in PwNT2. For PwIH, 3–14% were receiving disability or sickness payments [30, 56]; of these, 1 study reported non-statistically significant higher rates in PwIH compared with controls (3% vs 0%) [30] (Table 3).

**Table 3:** Summary of PwUN, PwNT1 and PwIH with disability benefits, support or holding “Disabled” status

	Author, year	Disability definition	Narcolepsy/IH cohorts	Control group	*p*-value vs. control
PwUN					
Disabled status or definition	Ferrans 1992 [38]	Disabled (unspecified)	9.7%	–	NA
	Flores 2016 [39]	Disability status	13.9%	8.5%	*p* = 0.004
	White 2020 [57]	Official recognition of disability	33.9%	4.8%	NR
	Abenza Abildúa 2023 [58]	Absolute permanent incapacity to work	7.4%	–	NA
Receiving disability payments	Broughton 1981 [28]	Disability insurance	10.6%	0%	NR
	Broughton 1978 [30]	Disability pension	16.0%	0%	NR
	Broughton 1984 [31]	Disability insurance	8.3%	0%	NR
	White 2020 [57]	Disability insurance pension	9.4%	1.4%	NR
	Kapella 2015 [45]	Disability benefit	9.8%	0%	NR
	Jennum 2020 [74]	Disability (unspecified)	10.5%	11.2%	*p* = NS
PwNT1					
Disabled status or definition	Peter-Derex 2024 [75]	Official recognition of disability	40.9%	5.6%	*p* < 0.0001
	Bassi 2024 [15]	Official recognition of disability	30.7%	5.3%	*p* < 0.001
	Alaia 1992 [27]	Consider themselves permanently disabled	15.0%	–	NA
Receiving disability payments	Kovalská 2016 [46]	Disabled pension before retirement	53.7%	13.0%	NR
	Peter-Derex 2024 [75]	Disability insurance pension	10.2%	3.8%	*p* < 0.0001
	Wasling 2020 [56]	Activity or sickness compensation	38.2%	–	NA
	Ingravallo 2008 [43]	Application for social benefits	47.0%	–	NA
PwIH					
Receiving disability payments	Broughton 1978 [30]	Disability pension attributed to IH	3.3%	0%	*p* > 0.5
	Wasling 2020 [56]	Activity or sickness compensation	14.3%	–	NA

*Abbreviations:* NA, not applicable; NR, not reported; NS, non-significant; PwIH, people with idiopathic hypersomnia; PwNT1, people with narcolepsy type 1; PwUN, people with unspecified narcolepsy type.

Two studies [15, 43] examined the degree of recognized disability in PwNT1 in Italy, where benefits are determined by Medical Commissions, who establish the percentage of disability proportionate to the reduction of work capacity. A higher percentage indicates greater disability, and therefore greater access to benefits; for example, 34–45% permits access to prostheses and aids, 46–73% provides employment support, 74–99% qualifies for a disability benefit, and 100% entitles the person to a full disability pension [43]. Bassi et al. reported that 31% of PwNT1 were officially recognized as disabled, most of whom had a degree of disability above the 46% cut-off for employment support (5% at 34–45%, 64% at 46–73%, 26% at 74–99%, and 5% at 100%) [15]. However, the percentage of recognized disability was shown to vary between Medical Commissions for identical patient cases, indicating that recognition is highly dependent on assessors’ interpretation [43].

### 4.6. Work productivity

#### 4.6.1. Absenteeism

Absenteeism on the Work Productivity and Activity Impairment (WPAI) questionnaire, expressed as the percentage of work time missed over the past 7 days due to their condition, was reported in 5 studies overall. Absenteeism ranged from 6% to 17% for PwUN [19, 39, 65], and was 5% for PwNT1 [15] and 12% for PwIH [18]. No studies reported absenteeism for PwNT2. Both case-control studies found significantly higher absenteeism among PwUN (17% vs. 7%, *p* < 0.001) [39] and PwNT1 (5% vs. 1%, *p =* 0.026) compared with controls [15] (Table 4). There were no comparisons of absenteeism between PwIH and a control group [15].

**Table 4:** Absenteeism and presenteeism on the Work Productivity and Activity Impairment questionnaire

Citation	Study design	Country	Absenteeism	Presenteeism	Overall work impairment
PwUN					
Flores 2016 [39]	CS (*n =* 189)	United States	PwUN: 17.4% Control: 7.3%	PwUN: 40.2% Control: 21.7%	PwUN: 45.5% Control: 24.8%
Emsellem 2020 [19]	RCT (*n =* 236)	United States/ Canada/Europe	PwUN: 6.1–14.9%	PwUN: 48.4–61.8%	PwUN: 61.5–70.7%
Weaver 2021 [65]	RCT (*n =* 186)	United States/ Canada/Europe	PwUN: 9.8%	PwUN: 58.9%	PwUN: 64.1%
Ohayon 2016 [48]	CS (*n =* 546)	United States			PwUN: 51%
PwNT1					
Bassi 2024 [15]	CS (*n =* 127)	Italy	PwNT1: 5.4% Control: 0.6%	PwNT1: 31.2% Control: 12.6% (*p* < 0.001)	PwNT1: 32.9% Control: 11.5%
PwIH					
Stevens 2023 [18]	CS (*n =* 75)	United States	PwIH: 12.3%	PwIH: 47.6%	PwIH: 51.4%

*Abbreviations:* CS, cross-sectional study; PwIH, people with idiopathic hypersomnia; PwNT1, people with narcolepsy type 1; PwUN, people with unspecified narcolepsy type; RCT, randomized controlled trial.

Four studies reported absenteeism in terms of the number of sick days per year. The way sick days are reported and the number of sick days reported as due to illness differ by country [76], and therefore the country where the study is based is included here. Averages of 13.6 and 15.8 sick days per year were reported for PwUN in 2 studies conducted in Germany [36, 47] and 7.6 sick days per year were reported in a US study compared with 3.0 days for controls (*p* < 0.0001) [59]. In one study conducted in France, PwNT1 had an average of 15.5 sick days per year versus 8.7 days for controls; however, the difference was not significant [75].

#### 4.6.2. Presenteeism

Presenteeism on the WPAI, or the percentage reporting that their condition affected productivity over the past 7 days, was reported in 5 studies. Presenteeism ranged from 40% to 62% for PwUN [19, 39, 65] and was 31% for PwNT1 [15] and 48% for PwIH [18] (Table 4). No studies reported presenteeism for PwNT2. Both studies with control groups found significantly higher presenteeism among PwUN (40% vs. 22%, *p* < 0.001) [39] and PwNT1 (31% vs. 13%, *p* < 0.001) compared with controls [15].

#### 4.6.3. Overall work impairment

Overall work impairment as captured by the WPAI, which includes both absenteeism and presenteeism, was reported by 6 studies (Table 4). WPAI ranged from 46% to 71% for PwUN [19, 39, 48, 65] and was 33% for PwNT1 [15] and 51% for PwIH [18]. In 2 of the 6 studies, overall work impairment was significantly higher with PwUN and PwNT1 than their respective control populations (46% vs. 25% for PwUN, *p* < 0.001 [39]; 33% vs. 12% for PwNT1, *p* < 0.001 [15]). No studies reported overall work impairment for PwNT2.

### 4.7. Career impact

#### 4.7.1. Career choices

Two studies reported that career decisions were impacted by symptoms of narcolepsy. In a survey in PwUN, 28% reported feeling unable to use their qualifications because of their narcolepsy [33]. In a second study of PwUN, 87% reported feeling restricted in the types of jobs to which they could apply for due to narcolepsy [68]. No data were identified for PwNT1, PwNT2, or PwIH.

#### 4.7.2. Occupational categories

One study comparing PwNT1 with controls reported no significant differences in the distribution of socio-professional categories between groups (*p =* 0.3) [75]. No data on occupational categories were identified for PwUN, PwNT2, or PwIH.

#### 4.7.3. Career progression

Seven studies reported that narcolepsy and IH can negatively affect career progression [28, 30, 31, 36, 38, 57, 70]. General impact on career progression was reported by 53% of participants in 1 study in PwUN [36], and 10–50% of PwUN reported that narcolepsy prevented a promotion across 5 studies [28, 30, 31, 38, 70]. Among PwIH, 1 study reported that 50% of participants perceived that IH symptoms had prevented a promotion compared with 0% of age- and sex-matched controls reporting the impact of any sleepiness on a promotion (*p* < 0.002) [30].

#### 4.7.4. Job change

One study reported the number of job changes among PwUN and 3 among PwNT1. No significant differences were observed in the number of job changes between PwUN (74% NT1, 26% NT2) and age- and sex-matched controls comprising friends and relatives [57]. Similarly for PwNT1, no significant differences in the number of job changes compared with controls were observed in 2 studies in which the control groups comprised acquaintances of the individuals with narcolepsy [75] and neighbors of the study authors [46]. In a third study, an NT1 diagnosis before the age of 30 years was significantly associated with fewer job changes (*p* < 0.05) [42]. In addition, a US survey found that 50% of participants with narcolepsy reported a perceived need for assistance in making career transitions [41]. No data on job changes were found for PwNT2 or PwIH.

#### 4.7.5. Work satisfaction

Different approaches for assessing work satisfaction were used across 4 studies: 2 in PwUN [53, 57] and 2 in PwNT1 [27, 75]. In 1 study of PwUN (51 NT1 and 18 NT2), participants reported significantly lower work satisfaction than age- and sex-matched healthy relatives (*p =* 0.03) [57]. Within specific domains, 30% of participants were satisfied with promotion prospects compared with 39% of controls (*p =* 0.07), and 53% reported satisfactory support in difficult situations compared with 75% of controls (*p =* 0.08) [57]. Another study reported that 47% of PwUN, most of whom were employed (86%), were significantly more dissatisfied with their work capacity than controls, as measured by the World Health Organization Quality of Life instrument [57]. However, the control group comprised hospital staff and volunteers with fixed daytime schedules, which may have influenced the comparison. In one study in PwNT1, participants experienced a significantly worse mean effort-reward imbalance at work relative to controls (*p =* 0.01), and 16% reported feeling misunderstood or perceived as lazy in the workplace [75]. A US survey of PwNT1 reported a mean overall job satisfaction score of 2.9 (standard deviation, 1.9) on a 5-point scale, with wide variability [27]. Greater satisfaction (higher score) was associated with jobs involving physical activity, those that allowed short naps, or those that permitted individuals to arrange their schedules to coincide with levels of alertness; however, no comparison group was included in this study [27].

#### 4.7.6. Job loss

Job loss (defined as losing or leaving a job) or unemployment due to narcolepsy or IH was reported by 10–52% of PwUN [28, 30–33, 35, 38, 42, 45, 54], 31–42% of PwNT1 [42, 44, 50], 25% of PwNT2 [50], and 23–26% of PwIH [30, 50].

Fear of job loss was reported by 15% to 49% of PwUN across 5 studies [28, 30, 31, 38, 70] and 33% of PwIH [30]. In the only study with a comparison group, significantly more PwUN feared job loss due to narcolepsy symptoms than controls feared job loss due to sleepiness (49% vs. 0%, *p* < 0.002) [28]. No data on fear of job loss were identified for PwNT1 or PwNT2.

#### 4.7.7. Income

Thirteen studies collected information on the impact of narcolepsy or IH on employment income: 9 for PwUN [28, 30, 31, 36, 38, 53, 61, 63, 74], 1 for PwNT1 [75], none for PwNT2, and 3 for PwIH [30, 60, 62].

Significantly reduced wages compared with age- and sex-matched control groups were reported in 4 studies with PwUN [28, 61, 63, 74], 1 study with PwNT1 [75], and 1 study with PwIH [60] (all *p* < 0.01). Additional studies involving PwUN reported marked reductions compared with healthy controls, based on descriptive analyses only and without statistical testing [30], and versus control groups comprised of people with epilepsy [31] or IH [30]. A further study in PwUN reported a non-significant reduction in income versus a control group comprised of hospital workers with fixed schedules [53]. Wage reductions as perceived by study participants were reported in a further 2 studies in PwUN (no comparison or control groups) [36, 38] and 1 study in PwIH [30], and income loss due to missed work time was reported in 1 study in PwNT1 [75]. One study reported no significant difference in income from employment between PwIH and controls [62].

### 4.8. Factors contributing to work-related outcomes

#### 4.8.1. Work-related problems

Many people with narcolepsy and IH report work-related problems arising from their condition. The most frequently reported problems by PwUN and NT1 included reduced performance (85%), being unable to stay awake/falling asleep at work (65–67%), work accidents (15–46%), and lateness (20–38%) [27, 43, 54, 57, 70, 75] (Table 5). One study reported that undefined work-related problems were experienced by 68% of 19 PwNT1 and 68% of 25 PwNT2 [40]. No data on work-related problems were found for PwIH.

**Table 5:** Work-related problems

Citation	Study design	Country	Patient-reported work-related problems	Patient-reported causes of work-related problems
PwUN				
White 2020 [57]	CS (*n =* 69)	France	Lateness: 37.9% (vs. 19.4% for controls)	NR
Geremew 2017 [40]	CS (*n =* 44)	Iran	Job problems (not defined): 68.2%	NR
Teixeira, 2004 [54]	CS (*n =* 49)	US	Falling asleep at work: 67% Accidents at work: 15%	NR
Rogers 1984 [70]	Qual CS (*n =* 20)	US	Unable to stay awake: 65% Sleeping during meetings: 20% Automatic behavior: 15% Negative reaction of coworkers: 15% Reprimanded for sleeping: 15% Sleep attacks compromising safety at work: 10%	NR
Jones 2016 [68]	Qual CS (*n =* 15)	US	Own uncertainty in good performance of job duties: 33% Coworkers' views of laziness or unproductivity: 27% Supervisor assuming use of the condition as excuse for underperforming: 7%	Sleep attacks/sleepiness: 100% Lack of focus: 100% Not alert: 80% Depression: 73% Extremely tired: 67% Muscle weakness: 20% Cataplexy: 20% Memory problems: 13% Hallucinations: 13% Negative reaction to medications: 7%
Broughton 1983, Broughton 1984 [29, 31]	CS (*n =* 60)	Canada	NR	Sleep attacks: 70.5% Poor concentration: 56.8% Poor memory: 31.8% Personality changes: 22.7% Interpersonal problems: 15.9%
	CS (*n =* 60)	Czechia	NR	Sleep attacks: 92.3% Poor concentration: 7.7% Poor memory: 0% Interpersonal problems: 3.8%
	CS (*n =* 60)	Japan	NR	Sleep attacks: 72.5% Poor memory: 45.1% Poor concentration: 41.2% Interpersonal problems: 37.3% Personality changes: 13.7%
Ferrans 1992 [38]	CS (*n =* 539)	US	NR	Sleep attacks: 63% Inability to concentrate: 40% Memory problems: 32% Personality changes: 15% Interpersonal problems: 14%
Broughton 1978 [30]	CS (*n =* 24)	Czechia	NR	Daytime sleepiness: 92%
PwNT1				
Peter-Derex 2025 [75]	CS (*n =* 235)	France	Lateness at work: Several times per week or month: NT1, 25.6%; controls, 8.2%; adjusted OR (95% CI), 4.49 (2.30–9.79) Several times per year: NT1, 20%; controls, 14.6%; adjusted OR (95% CI), 1.84 (1.04–3.27) Never: NT1, 54.4%; controls, 77.2%	Sleepiness: 59.2% Attention deficit: 23.9% Cataplexy: 8.5% Sleep inertia: 7.5%
Alaia 1992 [27]	CS (*n =* 102)	US	Reduced job performance due to narcolepsy: 85%	NR
Geremew 2017 [40]	CS (*n =* 19)	Iran	Job problems (not defined): 68.4%	NR
Ingravallo 2008 [43]	CS (*n =* 15)	Italy	Work accidents: 46%	NR
PwNT2				
Geremew 2017 [40]	CS (*n =* 25)	Iran	Work problems (not defined) 68.0%	NR
PwIH				
Broughton 1978 [30]	CS (*n =* 30)	Czechia	NR	Sleepiness (92%)

*Abbreviations:* CI, confidence interval; CS, cross-sectional study; NR, not reported; NT1, narcolepsy type 1; OR, odds ratio; PwIH, people with idiopathic hypersomnia; PwNT1, people with narcolepsy type 1; PwNT2, people with narcolepsy type 2; PwUN, people with unspecified narcolepsy type; Qual, qualitative; US, United States.

Patient-reported causes of work-related problems associated with narcolepsy included sleepiness or sleep attacks (59–100%), problems with concentration or focus (8–100%), and memory problems (0–32%) [29–31, 38, 68, 75] (Table 5). In one study of PwNT1, sleepiness (59%), attention deficit (24%), cataplexy attacks (9%), and sleep inertia (8%) were reported as the most disabling symptoms in the workplace [75].

#### 4.8.2. Workplace accommodations

Data from qualitative interview studies found that PwUN adopt a variety of work accommodations. These accommodations, such as provision to take naps and autonomy over individuals’ own work schedules, were reported by some PwUN [57, 68, 70], PwNT1 [50, 75], PwNT2 [50], and PwIH [50] as means to help them cope with their condition in the workplace. Switching tasks when feeling drowsy or avoiding tasks that require alertness were reported by 2 interview-based studies [68, 70]. Support/accommodation at work was reported by 21% of PwNT1 in one study, which consisted of schedule adaptation (52%), providing a time and a place for naps (59%), or allowing flexible organization (57%) and work from home (33%). A total of 36% of PwNT1 had a specific place for napping compared with 13% of age- and sex-matched controls (*p* < 0.0001) [75].

However, workplace accommodations can only be made when an employer has been made aware of the need, and very few studies report the proportion of people with narcolepsy who disclose their condition to their employer. One study reported that 24% of PwUN had concerns about disclosing their narcolepsy diagnosis to their employer, although the proportion who did or did not inform their employer was not reported [45]. A second study reported that 38% of PwUN had informed their employer about their condition, 38% had informed their direct line manager and 48% had informed the occupational health physician [57]. In a third study, 53% of participants with narcolepsy did not disclose their condition to their employer [68].

#### 4.8.3. Coping strategies

Coping strategies were reported by PwUN and PwNT1 in 3 cross sectional studies and 2 qualitative interview studies as means to help them cope with their condition in the workplace, including those intended to promote alertness, such as taking naps during work hours, consuming high levels of caffeine, splashing their face with water and physical movement, as well as those intended to hide their symptoms, such as pretending to be hyperactive, and avoiding meetings and examinations [50, 57, 68, 70, 75]. Other strategies reported by PwUN included avoiding getting overwhelmed, eating well while at work, exposing oneself to bright light, playing a computer game, smoking a cigarette, chewing ice, listening to music, and staying hydrated [68]. No other studies in PwNT2 or PwIH were found that reported data on coping strategies.

### 4.9. Factors correlating with work-related outcomes

#### 4.9.1. Symptom severity

The association between severity of narcolepsy and work productivity was assessed in 6 studies: 2 in PwUN [55, 66] and 4 in PwNT1 [15, 27, 42, 75] (Table 6). No data were reported for PwNT2 or PwIH. More severe EDS was associated with greater work impairment in 2 studies in PwUN and 1 study in PwNT1. In a 12-week RCT of solriamfetol in 231 PwUN (52% with NT1), improvements in sleepiness, assessed by change in Epworth Sleepiness Scale (ESS) total score at week 12, a widely used measure of the likelihood of falling asleep in sedentary or unstimulating situations, was highly correlated with reduced presenteeism (r = 0.527; *p* < 0.001) and overall work impairment (r = 0.503; *p* < 0.001), but correlations between ESS and WPAI absenteeism was low (0.246, *p* = NS). Correlations between MWT and WPAI scores were also low, between 0.143 and 0.268 (Table 6) [66]. In a cross-sectional survey of PwUN, severity of narcolepsy, measured using both the Ullanlinna Narcolepsy Scale (r = 0.29; *p* < 0.001) and the ESS (r = 0.25; *p* < 0.001), showed statistically significant but weak (i.e., r < 0.3) positive correlations with the “work dysfunction” domain of the Sickness Impact Profile (Table 6) [55]. For PwNT1, one study found ESS was strongly positively correlated (i.e., r between 0.5 and 0.7) with work impairment (r = 0.51; *p* < 0.001) and moderately positively correlated (i.e., r between 0.3 and 0.5) with missed work time (r = 0.35; *p* = 0.012), whereas no significant correlations were observed in the control group [15].

**Table 6:** Correlations between work outcomes and measures of symptom severity

	Clinical severity measure	Work outcomes	Correlation coefficient (*p*-value)
PwUN			
Weaver 2021 [66]	ESS (Pearson correlation coefficients)	Activity impairment Presenteeism Work impairment Absenteeism	0.436 (*p* < 0.001) 0.527 (*p* < 0.001) 0.503 (*p* < 0.001) 0.246 (*p* = NS)
	MWT (Pearson correlation coefficients)	Activity impairment Presenteeism Work impairment Absenteeism	−0.215 (*p* = NS) −0.268 (*p* = NS) −0.249 (*p* = NS) −0.143 (*p* = NS)
Ton 2014 [55]	UNS (Partial correlation coefficients)	Sickness Impact Profile (Work dysfunction)	0.25 (*p* < 0.001)
	ESS (Partial correlation coefficients)	Sickness Impact Profile (Work dysfunction)	0.29 (*p* < 0.001)
PwNT1			
Peter-Derex 2025 [75]	NSS	Recognition of disability	(*p* = 0.01)
Alaia 1992 [27]	4-point symptom severity scale (Pearson correlation coefficient)	Job satisfaction	0.13 (*p* = NS)
Bassi 2024 [15]	ESS (Spearman correlation coefficients)	Work time missed	0.35 (*p* = 0.012)
		Impairment while working	0.51 (*p* < 0.001)
		Overall work impairment	0.54 (*p* < 0.001)
			**Sex-adjusted OR (95% CI)**
Ingravallo 2012 [42]	Unemployed or inactive	Irresistible sleepiness	
		Onset before 30 years	1.5 (0.38–6.14)
		Diagnosis before 30 years	1.4 (0.37–5.73)
		Uncontrolled cataplexy	
		Onset before 30 years	2.8 (0.92–8.88)
		Diagnosis before 30 years	3.1 (0.97–9.61)
	Loss of working days	Irresistible sleepiness	
		Onset before 30 years	3.3 (1.04–10.6)^*^
		Diagnosis before 30 years	3.2 (1.08–9.61)^*^
		Uncontrolled cataplexy	
		Onset before 30 years	0.55 (0.14–2.12)
		Diagnosis before 30 years	0.47 (0.13–1.72)

Correlations with absolute values of 0–0.3 are considered low; absolute values of 0.3–0.5 are considered moderate; absolute values of >0.5 are considered high. No adjustments for multiplicity were made; therefore, *p*-values are nominal.

^*^ *p* < 0.05.

*Abbreviations:* ESS, Epworth Sleepiness Scale; NS, non-significant; NSS, Narcolepsy Severity Scale; OR, odds ratio; PwNT1, people with narcolepsy type 1; PwUN, people with unspecified narcolepsy type; UNS, Ullanlinna Narcolepsy Scale.

Other assessments of symptom severity reinforced these findings (Table 6). In a study of 100 working-age PwNT1, irresistible sleepiness significantly increased the odds of lost workdays (odds ratio, 3.2–3.3; *p* < 0.05) [42], and in a survey of 235 PwNT1, greater disease severity as measured by the Narcolepsy Severity Scale was significantly associated with formal recognition of disability status (*p =* 0.01) [75]. A small, non-significant correlation between assessment of symptom severity using a 4-point scale and job satisfaction was reported in a cross-sectional survey of PwNT1 [27].

#### 4.9.2. Health-related quality of life

One study in PwUN found that employment status was a significant predictor of HRQoL across most dimensions on the 36-Item Short Form Survey and on the EuroQol 5-dimension visual analog scale [35]. However, no studies were identified that explored the relationship between HRQoL and work-related outcomes in PwNT1, PwNT2, or PwIH.

#### 4.9.3. Narcolepsy treatment

Five studies reported the impact of narcolepsy treatment on work productivity. In PwUN, solriamfetol was associated with WPAI improvements in two, 12-week RCTs plus a long-term extension of one RCT. In the first trial (51% with cataplexy), all 3 solriamfetol doses produced reductions in presenteeism, overall work impairment, and activity impairment compared with placebo, reaching statistical significance for the 150-mg dose group, although absenteeism remained largely unchanged [19]. In an RCT long-term extension, mean (SD) percent improvements of 30% (25.5) in presenteeism and 30% (28.8) in overall work impairment in solriamfetol-treated PwUN were observed from baseline in the parent study to week 40 of the long-term extension (week 52 overall) [65]. In a cross-sectional study of PwNT1, stimulants, sodium oxybate, or venlafaxine had no impact on unemployment or absenteeism [42]. Finally, low sodium oxybate was associated with improvements in absenteeism, presenteeism, overall work impairment, and activity impairment (statistical significance not reported) in an RCT in PwIH and its open label extension study (4 and 6 months in duration) [17, 67].

One study compared work outcomes for treated vs. untreated individuals [50]. In PwNT1, 76% of treated and 75% of untreated participants were in paid work, and 59% of treated and 61% of untreated participants worked full-time. Among PwNT2, 92% of those receiving treatment were in paid work compared with 78% untreated, rising to 100% and 94%, respectively, when including students and homemakers; 83% of treated PwNT2 worked full-time versus 56% untreated, and part-time work was reported by 8% and 22%, respectively. For PwIH, full-time employment was 83% among treated and 68% untreated, with part-time work reported in 7% and 6%, respectively.

## 5. Discussion

This systematic review summarizes the available evidence on work-related outcomes among people with narcolepsy and IH. Current studies suggest reduced employment and increased disability in people with narcolepsy and IH, although fewer than half of the studies with control groups found significant differences. Limited available evidence indicates significantly greater absenteeism, presenteeism, and overall work impairment for people with narcolepsy and IH compared with controls. Individuals frequently reported feeling restricted in the types of jobs they could pursue, and some perceived negative effects on career progression, although socio-professional status appeared unaffected. Job change rates were comparable with controls, but about one-quarter reported losing or leaving a job because of their condition. Many described lower work satisfaction, effort-reward imbalance and feelings of being misunderstood, with some expressing a fear of job loss. Several studies reported reduced income among people with narcolepsy and IH, with approximately half reaching statistical significance compared with controls without a sleep disorder. Workplace difficulties were primarily attributed to EDS, impaired attention and cataplexy (NT1), as well as limited workplace accommodation and awareness. EDS severity was consistently linked to work outcomes, which in turn strongly influenced quality of life.

Substantial heterogeneity across studies limits the generalizability of existing evidence. Definitions of key outcomes, measurement tools, and reporting formats differed considerably, complicating synthesis and comparison. Few studies included control groups, and those that did often relied on suboptimal controls like friends, relatives or hospital staff, introducing bias. Data sources ranged from population registries to small convenience samples and advocacy surveys, often with low or unreported response rates, further limiting generalizability. Studies spanned several decades and countries, affecting comparability due to differences in labor markets, social policies, and treatment access. Diagnostic approaches were inconsistent, and many studies did not specify narcolepsy type. Characteristics such as disease duration, diagnostic delay, and current treatment varied widely, and few studies accounted for demographic, psychosocial, or occupational factors that may influence work outcomes. These limitations highlight the need for more rigorous, standardized, and representative research to inform clinical and policy decisions.

Our findings have clear implications for clinical practice despite limitations in the available evidence. Employment among people with narcolepsy or IH should not be interpreted as evidence of adequate functioning. Many individuals remain employed only through considerable personal effort, often also accepting restricted career options, diminished fulfilment, and the sacrifice of other life goals [4, 77]. Evidence suggests that individuals experience high residual symptom burden [78] and persistent work-related impairment despite treatment with currently available medications [17, 19, 42, 65, 67], suggesting that current therapies may not fully meet patient needs. However, the small number of studies and limited range of evaluated medications and treatment combinations prevent meaningful interpretation. Residual sleepiness, fatigue, and impaired concentration can lead to extended working hours, overcompensation, and an increased risk of burnout [4, 79]. Stigma and fear of discrimination further compound work challenges by discouraging disclosure to request accommodations [57, 77], which are often inconsistently granted when sought. Clinicians should ask questions about what patients are unable to do that matters to them. They should support requests for accommodations if desired and help educate employers and families about the disease, being mindful of the disbelief and stigma patients commonly encounter [13, 80]. Clinicians can also help by acknowledging where current treatments fall short and advocating for access to more effective medicines.

This review also has important implications for public policy and reimbursement decision-making, especially given the emergence of symptoms during critical developmental periods. Although work-related impairment, including absenteeism and presenteeism, among people with narcolepsy and IH contributes to substantial productivity losses, the most profound consequence of these disorders may be the underachievement of individual potential. Long diagnostic delays, inadequate treatment, and limited workplace support frequently alter educational and career trajectories, leading to decades of lost opportunities [11, 81]. This loss is particularly striking given the evidence that people with narcolepsy have been shown to demonstrate exceptional creativity compared with peers [82, 83]. Such underutilized potential represents a notable societal cost that is difficult to capture. Emerging evidence that effective treatment may improve work-related outcomes [17, 19, 65, 67] underscores the need to consider both the clinical and broader societal value of therapies when assessing treatment options. Practices that promote earlier diagnosis and access to effective treatment, along with measures that foster more supportive work environments are essential for reducing these long-term personal and societal losses.

Strengths of this systematic review were that it applied a comprehensive and structured approach, following PRISMA and Cochrane guidance, with clearly defined eligibility criteria, broad database coverage, and inclusion of multiple study designs to capture a wide range of work-related outcomes. Although the review was not registered in a public database, which may affect transparency, we strived to maintain methodological rigor throughout. Dual independent screening, validated critical appraisal tools, and detailed outcome specification strengthened the methodological rigor. However, notwithstanding the comprehensive searches and literature identification process, there is a risk that relevant studies may have been missed. Additionally, the handling of grey literature and reporting bias was not fully systematic. Due to heterogeneity among studies, a narrative synthesis approach was adopted. These factors should be considered when interpreting the findings.

This study was funded by Takeda and several authors are employees of the sponsor. To minimize the potential for bias associated with this relationship, the review followed a pre-specified methodology based on Cochrane guidance and was reported according to PRISMA recommendations. Two researchers from an external research organization independently conducted study selection, data extraction, and quality assessment using predefined criteria. All included data were obtained from publicly available, peer-reviewed sources. Interpretation of findings was based on the totality of the evidence and efforts were made to transparently report study limitations and heterogeneity across the evidence base. All authors critically reviewed the manuscript and approved the final version for publication.

Future research should establish and apply standardized definitions, validated measures, and patient-relevant concepts of workforce participation, productivity, and career impact to enable comparability across studies, settings, and time. Longitudinal studies incorporating both subjective and objective assessments are needed to clarify the causal pathways between narcolepsy or IH and work-related outcomes and the impact on quality of life. Building on this understanding, research should examine how time to diagnosis, symptom severity, treatment effects, social support, public education, and workplace accommodations influence these trajectories. Improved methods for assessing disability in these sleep disorders are also required to ensure equitable and appropriate recognition of impairment, as is research into how systemic factors such as labor laws, disability criteria, and workplace policies shape outcomes for people living with narcolepsy and IH. Finally, it is critical that people living with these diseases are actively involved in shaping the research, policy, and investment decisions that determine their futures.

## Supplementary Material

Supplementary-material_zpag078

## Data Availability

All data analyzed in this study are derived from previously published sources, which are cited in the reference list. No new data were generated or collected during this study. The study protocol was not registered in any database of systematic reviews.
