# Supplementary material for: The impact of narcolepsy and idiopathic hypersomnia on work-related outcomes: a systematic literature review
Source: Sleep Adv. 2026 Jul 17;7(3):zpag078. doi: 10.1093/sleepadvances/zpag078 (PMC13521521; doi:10.1093/sleepadvances/zpag078)
Supplement: Supplementary file 1 [file supplementary-material_zpag078.zip › Work Productivity SLR Supplement_13July2026_zpag078.docx]

**Supporting Information**

**The Impact of Narcolepsy and Idiopathic Hypersomnia on Work-Related Outcomes: A Systematic Literature Review**

Somraj Ghosh^1^, Stephen Crawford^1^, Sarah L. Bermingham^2^, Miriam L. Zichlin^1^, Fiona Stewart^3^, Nicole Fusco^3^, Evelyn Gomez-Espinosa^3^, Kristin Kistler^3^

^1^Takeda Development Center Americas, Inc., Cambridge, MA, United States, ^2^Takeda Pharmaceuticals USA, Inc., Cambridge, MA, United States, ^3^Cencora Global Consulting Services, Conshohocken, PA, United States

Corresponding author: Stephen Crawford, Takeda Development Center Americas, Inc., 500 Kendall Street, Cambridge, MA 02142, USA. Email: stephen.crawford@takeda.com.

**Supplementary Figure S1.** Summary of methodological quality assessment. (a) Cross-sectional studies (Joanna Briggs Institute Appraisal). (b) Qualitative studies. (c) Retrospective cohort studies (Motheral scale). (d) Prospective cohort studies (Newcastle-Ottawa Scale). (e) Randomized controlled trials (Cochrane risk-of-bias tool).

**Supplementary Table S1.** Study inclusion and exclusion criteria.

| **PICOS** | **Inclusion criteria** | **Exclusion criteria** |
| --- | --- | --- |
| Population | Humans of all ages with narcolepsy (with or without cataplexy) or IH | Not human  No narcolepsy or IH |
| Intervention | No limits | No limits |
| Comparator | No limits | No limits |
| Outcomes | - Employment - Unemployment - Absenteeism - Presenteeism - Number of days/hours - Overall work impairment - Fear of job loss - Actual job loss or leaving - Disability leave/permanent disability/permanent incapacity - Early retirement - Income - Socio-professional categories - Job changes - Career progression - Career choices - Work satisfaction - Accommodations/coping strategies - Work-related problems - Patient-reported causes of work productivity impairments | No outcomes related to work productivity |
| Study design | - Observational (e.g., retrospective and prospective cohort studies, cross-sectional studies/surveys, case-control studies) - Qualitative studies (e.g., patient interviews, focus groups, social media listening) - Experimental/interventional (e.g., RCTs and non-randomized trials, including withdrawal trials; single-arm intervention studies) - SLRs/meta-analyses/TLRs/narrative/ scoping literature reviews^a^ | Case studies (i.e., studies explicitly stated to be “case studies” and/or studies with <10 participants)  Other than included study designs |

^a^Published literature reviews were tagged for manual reference checks but excluded from the list of final included studies.

*Abbreviations:* IH, idiopathic hypersomnia; PICOS, population, intervention, comparator, outcome, study design; RCT, randomized controlled trial; SLR, systematic literature review; TLR, targeted literature review.

**Supplementary Table S2.** Search strategy.

| **EBM Reviews - Cochrane Central Register of Controlled Trials <January 2024>**; **EBM Reviews - Cochrane Database of Systematic Reviews <2005 to February 28, 2024>**; **Econlit <1886 to February 22, 2024>**; **Embase <1974 to 2024 March 01>**; **Ovid Emcare <1995 to 2024 Week 08>**; **Ovid MEDLINE(R) ALL <1946 to March 01, 2024>** | | |
| --- | --- | --- |
|  | **Search string** | **Number of hits on 5 March 2024** |
| 1 | exp "disorders of excessive somnolence"/ | 35,268 |
| 2 | exp Hypersomnolence/ | 21,007 |
| 3 | exp Narcolepsy/ | 16,539 |
| 4 | exp Idiopathic Hypersomnia/ | 1245 |
| 5 | (hypersomn$ or narcolep$ or cataplex$ or gelineau or (narcolep$ adj2 cataplexy) or (sleep adj (epilepsy or paroxysmal))).mp. | 31,226 |
| 6 | or/1-5 | 45,434 |
| 7 | exp Productivity/ | 104,493 |
| 8 | teleworking/ | 2265 |
| 9 | exp Presenteeism/ | 3763 |
| 10 | exp Absenteeism/ | 38,088 |
| 11 | ((work$ or telework$ or school$ or academ$ or university or education$ or employ$ or job$) adj3 (activit$ or perform$ or career$ or productivit$ or impair$ or problem$ or inabilit$ or disabilit$ or progress$ or limit$ or influenc$ or impact$)).tw,kw. | 667,270 |
| 12 | (unemployment or ((disabilit$ or sick$ or illness) adj3 (leave or pay$)) or presenteeism or absenteeism or ((out or reduc$ or loss$ or decrease$ or prevent$ or avoid$ or dismiss$) adj3 (job$ or work$ or employ$ or profession$ or income or salary or promot$ or progress$ or advancement))).tw,kw. | 710,828 |
| 13 | (work productivity or activity impairment or early retirement or wpai or sickness impact profile).tw,kw. | 22,193 |
| 14 | Socioeconomics/ or Socioeconomic/ | 192,991 |
| 15 | (socioeconomic$ or socio-economic$ or (psychosocial adj (impact or effect$ or burden))).tw,kw. | 537,268 |
| 16 | ((job? or work$) adj3 (schedule or hour$ or relation$)).tw. | 120,721 |
| 17 | (employment or academic$).ox. | 153,550 |
| 18 | or/7-17 | 2,221,146 |
| 19 | 6 and 18 | 2085 |
| 20 | exp animals/ not exp humans/ | 10,704,649 |
| 21 | (rat or rats or mouse or mice or murine).ti. or (nonhuman or in vitro study or in vivo study).hw. | 11,329,966 |
| 22 | (editorial or comment$ or note or study guide).pt. | 3,997,521 |
| 23 | case study/ or editorial/ or case report/ or news/ or note/ or study guide/ | 8,775,361 |
| 24 | or/20-23 | 26,336,188 |
| 25 | 19 not 24 | 1804 |
| 26 | remove duplicates from 25 | 1389 |

**Supplementary Table S3.** Characteristics of included studies.

| **References** | **Study design** | **Country** | **Data source (study years)** | **Narcolepsy/IH case definition** | **Sample size** | **Female** | **Age, mean (SD), years** | **Current narcolepsy medications** | **Controls** | **Strengths or limitations** |
| --- | --- | --- | --- | --- | --- | --- | --- | --- | --- | --- |
| [1] | RC | US | Truven Health Analytics MarketScan Research Databases (2006–2010) | Diagnosis code of narcolepsy +/- cataplexy according to ICD-9 | Overall: 9312  With productivity data: 600 | Overall: 59.2%  With productivity data: 38% | Overall: 46.1 (13.3)  With productivity data: 41.1 (NR) | NR | 46,559 without narcolepsy diagnosis, randomly selected then matched by age, sex, 1 of 4 US geographic regions and payer (Commercial or Medicare) | Broadly population based; quality control group |
| [2]  (abstract) | PC | US | Survey of patients identified via online communities and patient panels who were diagnosed with narcolepsy taking Sodium oxybate for ≥12 months (NR) | NR | 102 | 88% | 36.8 (9.9) | Sodium oxybate: 102 (100%) | No controls | Unknown response rate; limited to patients treated with Sodium oxybate for ≥1 year |
| [3] | CS | US | Survey sent to ANA mailing list (dates NR) | NR | 539 | 56.2% | 52.6 (12.8) | Respondents "were being treated medically" but does not report any further details | No controls | Large sample, low response rate (68.8%); states included may not represent all US; older data |
| [4] | CS | US | NHWS (2011–2013) | Individuals who report having been diagnosed with narcolepsy by a physician | 437 | 50.1% | 46.7 (16.4) | NR | 837 propensity-matched controls without narcolepsy | Large sample, US-representative; propensity matched to controls; limitations: self-reported narcolepsy diagnosis, older data |
| [5] | CS | US | Consecutive patients from Sleep-Wake Disorders Center at Montefiore Medical Center who responded to mailed questionnaires (1986) | NR | 68 | 47% | 46 (11.7) | NR | No controls | Single center; convenience sample; low response rate (57%); older data |
| [6] (dissertation) | Qual CS | US | Semi-structured interviews with people (≥18 years diagnosed with narcolepsy and employed for ≥3 years in an office, clerical, health care or educational setting) identified via social media and professional contacts of employees (2015–2016) | Self-reported diagnosis of narcolepsy | 15 | 60% | Median 40 [IQR 29–51] | NR | No controls | Small sample, unknown representativeness |
| [7] | CS | US | Young adults (18–37 years) with narcolepsy who contacted the University of Illinois at Chicago, Center for Narcolepsy Research, asking to be placed on the mailing list for research participation (dates NR) | Self-reported diagnosis of narcolepsy | 122 | 77.9% | 27.1 (5.0) | NR | An acquaintance approach was used to obtain a control group of young adults without narcolepsy | Self-selected participants; young adults (not generalizable to all patients with narcolepsy); control group were acquaintances |
| [8] | CS | US | Survey distributed via Unite Narcolepsy website and other online channels (2013) | Self-reported diagnosis of narcolepsy | NT1: 997  NT2: 329  Did not know if NT1 or NT2: 149  Total: 1697 | NR | 17: 8.5%  18–34: 47.5%  35–54: 37.5%  55–74: 6.5% | Armodafinil: 231/1103 (20.9%)  Amphetamine: 360/1118 (32.2%)  Modafinil: 348/1199 (29%)  Sodium oxybate: 422/1199 (35.2%) | No controls | Self-reported narcolepsy; unclear response rate (self-selection bias could influence study results) |
| [9] | Qual CS | US | Semi-structured interviews; recruited from Greater Chicago Area Narcolepsy Association (*n* = 14) and a psychologist associated with a local sleep disorder center (dates NR) | NR | NT1: 16  NT2: 4  Total: 20^a^ | 55.0% | Range 27–68 | Stimulants: 17 (85%)  Tricyclics: 2 (10%)  None: 3 (15%) | No controls | Small, selected sample; older data based on publication date |
| [10] | CS | US | Survey administered to patients in King County, Washington; attempted to locate all patients in King County; US (dates NR) | NR | NT1: 152  NT2: 74  Total: 226^a^ | NR | 48 (NR) | NR | No controls | Response rate 66% |
| [11] | CS | US | Focus group and survey (2018) | "documented diagnosis of narcolepsy (Type I or Type II)" (no further details reported) | NT1: 17  NT2: 12  Total: 29 | 93.1% | 31.1 (7.6) | NR | No controls | Small sample; inclusion criteria included elevated symptoms of depression defined as a total score of ≥10 on the PHQ-9; the primary method of recruitment was through online resource |
| [12] | CS | BR | EDS outpatient clinic in São Paulo (2020) | Diagnosis of NT1 or NT2 according to the American Academy of Sleep Medicine guidelines | NT1: 52  NT2: 24  Total: 76^a^ | 68.4% | 36.9 (11.7) | Stimulant: 34.2%  Antidepressant: 5.3%  L-carnitine: 1.3%  Stimulant + antidepressant: 5.3%  Stimulant + L-carnitine: 1.3%  Stimulant + antidepressant + L-carnitine: 2.6%  None: 7.9% | No controls | Unclear generalizability of sample or response rate |
| [13] | CS | BR | Neurology department outpatient clinic (dates NR) | ICSD | 40 | 70% | 41.8 (14.5) | NR | Hospital workers or volunteers with fixed daytime work schedules presenting no chronic diseases or sleep disorders | No information on how patients were selected (unclear generalizability) |
| [14] | CS | CAN | Survey; Epilepsy Clinic, Ottawa General Hospital; presumably part of the 180 in Broughton, 1981 | History of more or less irresistible sleep attacks and cataplexy with or without the other auxiliary symptoms of sleep paralysis | 60 | 55% | 41.4 (11.1) | Methylphenidate: 25 (41.7%)  Amphetamine: 13 (21.7%)  Tricyclic antidepressants: 20 (33.3%)  Monoamine oxidase inhibitor: 1 (1.7%) | 60 patients with epilepsy (matched on duration of disease) | Single center |
| [15] | RC | DK | NPR | Diagnosis of narcolepsy in the secondary sector and in public and private hospitals | 462 | 52.2% | NR | NR | Randomly selected 1836 age- and sex- matched citizens without narcolepsy/other hypersomnias | Representative of all patients in DK with narcolepsy diagnosis in the primary or secondary sector in both public and private hospitals; limitation: older data |
| [16] | RC | DK | NPR (1997–2009) | Diagnosis of narcolepsy in the secondary sector and in public and private hospitals | 816 | 54% | <20: 10%  20–29: 19%  30–39: 15%  40–49: 12%  50–59: 17%  60–69: 13%  70–79: 8%  >80: 5% | NR |  |  |
| [17] | PC | DK | NPR (1994–2015) | Diagnosis of narcolepsy in the secondary sector and in public and private hospitals, before age 18 years (status at age 20 years) | 171 | 46.2% | At diagnosis: 15.2 (3.4); comparison was made with controls at age 20 years | NR | Randomly selected 680 age- and sex- matched individuals living in the same municipality, without narcolepsy/other hypersomnias | Representative of all patients in DK with narcolepsy diagnosis at age <18 years in the primary or secondary sector in both public and private hospitals |
| [18] | CS | FR | Patients >18 years, usually followed up at the Lyon Sleep Medicine Center and diagnosed with narcolepsy, were solicited by regular mail for the study (2017–2018) | ICSD-3 | NT1: 51  NT2: 18  Total: 69 | 50.7% | 42.5 (18.2) | Stimulants (1 drug, 2 drugs, ≥3 drugs): 91.3% (75.4%, 11.6%, 4.3%)  Modafinil: 67%  Methylphenidate: 29%  Pitolisant: 19%  Dexamphetamine: 0.01%  Anti-cataplectic drugs: 54%  Antidepressant drugs: 38%  Sodium oxybate: 17% | The control group consisted of 1 or 2 age- and sex-matched (±5 years, age >18 years) relatives of the patients (mainly friends or cousins) without narcolepsy | Single center |
| [19] | RC | DE | InGef research database (2014–2018) | ≥2 claims in the database that included diagnoses of narcolepsy in 2 different quarters, ICD-10-GM Code G47.4 | 413 | 45.2% | 50 (SD not reported) | NR | No controls | DE population based; inclusion criteria required ≥1 pharmaceutical treatment for EDS; thus, untreated patients not included |
| [20] | CS | DE | Regional statutory health insurance fund AOK PLUS [claims database] (2013–2018) | ≥2 claims in the database that included diagnoses of narcolepsy in 2 different quarters, ICD-10-GM code G47.4 | 133 | 36.3% | 46.23 (18.56) | Modafinil: 24.1%  Sodium oxybate: 9.0%  Venlafaxine: 6.8%  Methylphenidate: 5.3%  Clomipramine: 4.5%  No narcolepsy-related medication: 59.4% | No controls | Broadly population based (only data from 1 regional DE sickness fund; the results may not be fully representative of the entire German population) |
| [21] and [22] | CS | DE | Single sleep center; all patients treated at Hephata Klinik (1999–2002) | ICSD | 75 | 38.6% | 48.9 (15.2) | NR | No controls | Moderate response rate; 75/102 participated in phone survey (74%); single center; older data |
| [23] | CS | Iran | Single sleep center (2010–2015) | ISCD-2 | NT1: 19  NT2: 25  Total: 44 | NT1: 21%  NT2: 16%  Total: 18.8% | NT1: 30.1 (11.4)  NT2: 35.6 (12)  Total: 33.2 (11.9) | NR | 50 healthy age- and sex- matched | Small sample; unknown representativeness or how cases were selected |
| [24] | CS | NZ | All Narcolepsy Support Group of NZ members and sleep physicians, neurologists and sleep laboratories throughout NZ (dates NR) | NR | 54 | 63% | 54.7 (18.3) | Any medication: 44 (81.5%)  Stimulant: 16 (29.6%)  Antidepressants: 10 (18.5%) | No controls | Moderate response rate (70%); unclear how representative of all people with narcolepsy |
| [25] | CS | PT | Survey sent to patients at 3 sleep centers (2006–2007) | ICSD | 51 | 49% | 43.4 (15.3) | NR | No controls | Participants from 3 Portuguese sleep centers; 51/70 invited (73%) participation rate; unclear if 70 represented all patients; unclear how representative the 3 sleep centers were |
| [26] | RC | ES | All patients attending Infanta Sofía University Hospital, Madrid (2012–2022) | NR | NT1: 27  NT2: 27  Total: 54^a^ | 57% | 40.5 (14.6) | NR | No controls | Single center; unclear how representative of all people with narcolepsy; recent data |
| [27] | CS | UK | Questionnaire sent to 500 randomly selected members of the UKAN [total membership of UKAN unknown at time of the study] (1997) | NR | 305 | 60.7% | 56 [range 18–89] | Stimulant and anti-cataplexy: 61 (20%)  Stimulant only: 148 (48.5%)  Anti-cataplexy only: 22 (7.2%)  Neither stimulant nor anti-cataplexy: 74 (24.6%)  Modafinil: 0 | No controls | Response rate low (63%) |
| [28] | CS | UK | Single sleep center (dates NR) | ICSD | 49 | 48.7% | 47.0 (18.0) | On treatment for their narcolepsy: 93.8%  Stimulants: 87.7%  Patients who had previously received therapy and opted to be managed by lifestyle alteration only: 6.1% |  |  |
| [29] and [30] | CS | CAN, CZ, JP | Survey; unclear how sampling was done; 60 from each center | NR | 180 | 38% | 41.9 (11.3) | Methylphenidate: 63 (35%)  Amphetamines: 21 (11.7%)  Ephedrine: 6 (3.3%)  Phenmetrazine: 6 (3.3%)  Dexphenmetrazine: 2 (1.1%)  Tricyclic drugs: 59 (32.8%)  MAO inhibitors: 2 (1.1%)  Hypnotics and tranquillizers: 31 (17.2%)  Not receiving any CNS-active drugs: 20 (11.1%) | 180 age- and sex- matched controls | Unclear how participants were identified/recruited; old data |
| [31] | RCT | US, CAN, FL, FR, DE, IT | NCT02348593, trial of solriamfetol versus placebo (dates NR) | ICSD-3 | 177 | 67.2% | Solriamfetol 75 mg: 36.5 (12.8)  Solriamfetol 150 mg: 38.1 (13.0)  Solriamfetol 300 mg: 34.3 (11.5) | None reported other than the study drug |  |  |
| [32] and [33] | CS | International (92% US) | Nexus Narcolepsy Registry (2015–2017) | Any diagnosis of narcolepsy by a doctor | NT1: 661  NT2: 322  Total: 983^a^ | 84.8% | 38.0 (95% CI, 37.4–38.6) | Of 1760 assessments (983 participants):   - No (additional) pharmacologic treatment: 187 (10.6%) - Any treatment: 1573 (89.4%) - Armodafinil or modafinil alone: 229 (13.0%) - Stimulants alone: 396 (22.5%) - Stimulants with armodafinil or modafinil: 141 (8.0%) - Antidepressants alone: 92 (5.2%) - Antidepressants + stimulants: 333 (18.9%) - Antidepressants + armodafinil or modafinil: 226 (12.8%) - Antidepressants + stimulants + armodafinil or modafinil: 156 (8.9%) | No controls | Large study, recent data; limitation: the registry population similar to that of the general US population in terms of geographic distribution and the proportion of participants with private health insurance; the registry population was predominantly female, White and highly educated —characteristics linked to a higher likelihood of survey participation |
| [34] | RCT (data analyzed regardless of group allocation) | EUR; NAM | NCT02348593 (dates NR) | NR | 231 | 64.9% | 36.2 (13.2) | NR |  |  |
| [35] | OLE of RCT | EUR; NAM | NCT02348632 (2015–2017) | NR | 278 | 64.6% | 38.7 (13.5) | Solriamfetol (study drug): 100% |  |  |
| [36] | CS | US | Surveys sent to randomly selected members of the ANA (dates NR) | Both EDS or sleep attacks and cataplexy symptoms | 102 | 59% | 53.5 (11) | Currently taking medications for some or all symptoms: 80 (78.2%)  For EDS: 19 (19%)  For EDS and cataplexy: 18 (18%)  For EDS and sleep attacks: 12 (12%)  For cataplexy: 4 (4%)  For restless nighttime sleep: 1 (1%)  For all symptoms: 14 (14%) |  |  |
| [37] | CS | US | Single sleep center “patients with both sleep attacks and cataplexy were selected from among patients seen in our Sleep Disorders Clinic” (dates NR) | NR | 50 | 56% | 42.6 [range 18–72] | NR | Unknown where controls were from (groups of age- and sex-matched control participants who had no past or current sleep disorders) | Unknown how many included out of all patients at center; older data |
| [38] | CS | CZ | Single sleep center, 42^[1]^ patients aged >60 years with NT1 at a tertiary hospital, in a sleep disorders center providing long-term care of hypersomnia patients, were invited to participate (2012–2015) | Adults aged >60 years fulfilling  ICSD-2 criteria for narcolepsy and undisputable cataplexy | 42 | 57.1% | 71.9 (7.5) | Stimulants: 26 (61.9%)  Antidepressants: 21 (50.0%)  Combination of stimulants and antidepressants: 15 (35.7%)  Sodium oxybate: 2 (4.8%) | 46 age- and sex-matched controls recruited from a leisure activity center for the elderly or were neighbors of the  3 co-authors | Single center; generalizable to older adults only |
| [39, 40] | CS | FR | 18 sleep centers, France (2020–2023) | ICSD-3 | 235 | 63.8% | 36.4 (14.7) | Modafinil: 93 (40.4%)  Pitolisant: 48 (20.9%)  Methylphenidate: 71 (30.9%)  Dexamphetamine: 2 (0.9%)  Venlafaxine: 52 (22.6%)  Fluoxetine: 8 (3.5%)  Clomipramine: 13 (5.7%)  Sodium oxybate: 38 (16.5%)  ≥1 wake promoting drug: 184 (80%)  ≥1 anti-cataplectic drug: 99 (43%)  ≥1 wake promoting and/or anti-cataplectic drug: 199 (86.5%) | 2 acquaintances (not relatives, same sex and age [±5  years]) willing to complete the same questionnaires except for those specific to narcolepsy | Response rate was very low (15%); a total of 1560 letters were sent to all patients with NT1 registered in the active files of the 18 participating centers in France; among them, 235 (63.8% women, age 36.4 ± 14.7 years) completed the questionnaires; authors note that the participants likely represented the least severe patients; the sex imbalance does not reflect the actual sex ratio of the disease; the employment rate and graduation level were lower in non-respondent than in respondent patients; strength = recent data (2020−2023) |
| [41] | CS | IT | Clinic for Narcolepsy of the Sleep Disorders Center of the University of Bologna (2009−2011); 100 consecutive working-age (18–65 years) patients with narcolepsy of ≥6 months (2009–2011) | ICSD-2 | 100 | 49% | 37.1 [range 18–65] | Treated patients (*n* = 79)  Stimulant only: 35 (44%)  Stimulant and anti-cataplexy: 13 (17%)  Sodium oxybate only: 8 (10%)  Sodium oxybate and stimulant: 10 (13%)  Sodium oxybate and anti-cataplexy: 5 (6%)  Sodium oxybate, stimulant and anti-cataplexy: 7 (9%)  Anti-cataplexy only: 1 (1%) | Italian National Census Bureau 2008 data for the same age range (18–65 years) | 100 consecutive working-age (18–65 years) patients with narcolepsy of ≥6 months from the Clinic for Narcolepsy of the Sleep Disorders Center of the University of Bologna; “consecutive patients” suggest a convenience sample—unclear how many patients were not included. Authors note: “a possible recruitment bias, as the Bologna Outpatient Clinic for Narcolepsy is one of the major centers for narcolepsy in Italy, thus probably attracting the more severe cases. Moreover, narcoleptic patients who did not come back for a follow-up visit, and presumably the less severe cases, were excluded”; older data (2009−2011) |
| [42] | CS | IT | The Bologna Sleep Center Narcolepsy database; 1 expert in sleep medicine and 1 specialist in legal medicine selected the patients with different disease severity from a database of 150 people with narcolepsy (2005) | ICSD-2 | 15 | 40% | 48.7 (18.8) | Modafinil: 7 (47%)  Modafinil + anti-cataplexy: 6 (40%)  No drug: 2 (13%) | No controls | Small sample size; potential selection bias (1 expert in sleep medicine and 1 specialist in legal medicine selected patients with different disease severity from a database of 150 people with narcolepsy); older data |
| [43] | CS | IT | Outpatients attending Narcolepsy Center of Bologna, Italy (dates NR) | ICSD-3 | 127 | 53.5% | 38.2 (15.5) | Stimulants: 68 (53.5%)  Sodium oxybate: 81 (63.8%)  Anti-cataleptic: 27 (21.3%)  Other drugs: 5 (3.9%)  None: 11 (8.7%) | Parents, children, partners or friends who accompany people with narcolepsy or other neurological disorders, matched for sex, age and educational level | No information on how the patients were selected/ participation rate (“Patients were recruited from those currently attending the outpatient clinic of the Narcolepsy Center of Bologna, Italy”) |
| [44] | CS | The Netherlands | Two sleep centers (dates NR) | ICSD-2 | 116 | 48.3% | 41.8 (15.4) | Using medication for cataplexy (either antidepressants or sodium oxybate): 65 (56.0%) | No controls | Response rate NR |
| [45] | CS | NOR | Surveys sent to members of the Norwegian Association for Sleep Disorders (2003) | ICSD | 77 | 70% | 53 (17.4) | Medication for EDS and cataplexy: 39%  Medication for EDS only: 36%  Neither for EDS nor for cataplexy: 25% | Normative QoL data | Moderate response rate: 78% |
| [46] | CS | US | Real World Idiopathic Hypersomnia Outcomes Study [ARISE] (2021) | Individuals who report having been diagnosed with IH by a physician | 75 | 81.3% | 34.1 (10.7) | Off-label medications for IH: 69 (89.3%) |  |  |
| [47] | CS | CZ | Prague neurological clinic (dates NR) | Roth criteria | 30 | 40% | 38.4 [range 20–66] | Phenmetrazine: 10 (33%)  Methylphenidate: 9 (30%)  Amphetamines: 2 (7%)  Dexphenmetrazine: 1 (3%)  Other: 4 (13%)  No medication: 4 (13%) | Age- and sex-matched to 24 patients with IH | Small sample; old data;  no information on sampling frame |
| [48] | RC | DK | NPR (1998–2005) | Diagnosis of hypersomnia in the primary or secondary sector in public and private hospitals | 2208 | 21.6% | NR (“most above 30 years of age”) | NR | 4 randomly chosen citizens with the same age and sex but no hypersomnia nor hypersomnia diagnosis | Population based; older data |
| [49] | RC | DK | NPR (1997–2009) | Diagnosis of hypersomnia in the primary or secondary sector in public and private hospitals | 2855 | 25.5% | <20: 49 (1.7%)  20–29: 243 (8.5%)  30–39: 551 (19.3%)  40–49: 725 (25.4%)  50–59: 796 (27.9%)  60–69: 396 (13.9%)  70–79: 74 (2.6%)  >80: 21 (0.7%) | NR | 11,382 randomly selected citizens who had the same age and sex as the patients and resided in the same area of the country in which the patients lived and who had the same marital status; the ratio of control participants to patients was 4:1 |  |
| [50] | OLE of RCT |  | NCT03533114;  Belgium, Czech Republic, Finland, France, Poland, Spain, US (dates NR) | ICSD-2 or ICSD-3 | 154 | 68% | 40.3 (13.7) | Sodium oxybate: 100% (study drug) |  |  |
| [51] | RCT |  | NCT03533114; North America and Europe (2018–2020) | ICSD-2 or ICSD-3 | 154 | 68.2% | 40.3 (13.7) | Sodium oxybate only: 2/154 (1.3%)  Sodium oxybate + alerting agent: 4/154 (2.6%)  Alerting agent only: 82/154 (53.2%)  Treatment naïve: 66/154 (42.9%) |  |  |
| [52] (abstract) | Qualitative | NR | Semi-structured interviews with patients recruited from a patient advocacy group and a rare disease registry  (country and dates NR) | NR | 20 | 70% | 35 (NR) | NR |  |  |
| [53] | CS | NR | Interview and survey to inform dynamic simulation model | ICSD-2 or ICSD-3 | 43 | NR | Median 43 [IQR 19.5] | NR |  |  |
| [54] | CS |  | Survey administered to patients at a sleep center, JP (2007–2009) | ICSD-2 criteria | NT1: 83  NT2: 48  IH: 54  Total: 185 | NT1: 48.2%  NT2: 47.9%  IH: 37.0%  Total: 44.9% | NT1: 33.2 (9.8)  NT2: 30.5 (6.0)  IH: 33.3 (7.0)  Total: 32.6 (8.3) | NT1:   - Stimulant and anti-cataplexy: 18 (21.7%) - Stimulant only: 65 (78.3%) - ≥2 stimulants: 35 (42.2%)   NT2:   - Stimulant and anti-cataplexy: 0 - Stimulant only: 48 (100%) - ≥2 stimulants: 14 (29.2%)   IH:   - Stimulant and anti-cataplexy: 0 - Stimulant only: 54 (100%) - ≥2 stimulants: 16 (29.6%)   Total:   - Stimulant and anti-cataplexy: 18 (9.7%) - Stimulant only: 167 (90.3%) - ≥2 stimulants: 65 (35.1%) |  |  |
| [55] | CS | SE | Outpatient clinic of the Department of Neurology, Sahlgrenska University Hospital in Gothenburg (2019–2020) | ICSD-3 criteria | NT1: 68  IH: 21  Total: 89 | NT1: 54.4%  IH: 71.4% | NT1: NR  IH: 43.0 (15.3) | NT1:   - Modafinil: 13 (19%) - Methylphenidate: 30 (44%) - Amphetamine: 7 (10%) - Dexamphetamine: 32 (47%) - Sodium oxybate: 22 (32%) - No stimulant: 12 (18%) - 1 stimulant: 42 (62%) - 2 stimulants: 14 (21%) - Antidepressants: 27 (40%)   IH:   - Modafinil: 4 (19%) - Methylphenidate: 5 (24%) - Amphetamine: 4 (19%) - Dexamphetamine: 4 (19%) - Sodium oxybate: NA - No wake promoting medication: 5 (24%) - 1 stimulant: 15 (71%) - 2 stimulants: 1 (5%) - Antidepressants: 2 (9.5%) | 23 age- and sex-matched healthy controls recruited from the local community; inclusion criteria were the ability to understand and provide informed consent, no history of neurological or psychiatric disorder and no current intake of medications known to alter sleep or cognition | 45 with NT1 after H1N1 vaccination (57.3% response rate); 23 with NT1 without H1N1 vaccination (62.2% response rate); 21 with IH (67.7% response rate) |

*Abbreviations:* ANA, American Narcolepsy Association; Aus, Australia; BR Brazil; CAN, Canada; CI, confidence interval; CNS, central nervous system; CS, cross-sectional study; CZ, Czechia; DE, Germany; DK, Denmark; EDS, excessive daytime sleepiness; ES, Spain; EUR, Europe; FL, Liechtenstein; FR, France; ICD-9, International Classification of Diseases, Ninth Revision; ICD-10-GM, International Classification of Diseases, Tenth Revision, German Modification; ICSD(-2/3), International Classification of Sleep Disorders (Second/Third Edition); IH, idiopathic hypersomnia; IQR, interquartile range; IT, Italy; JP, Japan; MAO, monoamine oxidase; NA, not applicable; NAM, North America; NHWS, National Health and Wellness Survey; NOR, Norway; NPR, National Patient Registry; NR, not reported; NT1, narcolepsy type 1; NT2, narcolepsy type 2; NZ, New Zealand; OLE, open-label extension; PC, prospective cohort study; PHQ-9, Patient Health Questionnaire-9; PT, Portugal; Qual, qualitative; RC, retrospective cohort study; SD, standard deviation; SE, Sweden; UK, United Kingdom; UKAN, Narcolepsy Association UK; US, United States.

**References**

1.	Black J, Reaven NL, Funk SE*, et al.* The Burden of Narcolepsy Disease (BOND) study: health-care utilization and cost findings. *Sleep Med.* 2014;**15**(5):522–529. <https://doi.org/10.1016/j.sleep.2014.02.001>.

2.	Bae C, Shah S, Profant J*, et al.* Sodium oxybate treatment regimen and dosing personalization among patients with narcolepsy in a real-world setting. *Sleep Med.* 2019;**64**(Supplement 1):S19. <https://doi.org/10.1016/j.sleep.2019.11.056>.

3.	Ferrans CE, Cohen FL, Smith KM. The quality of life of persons with narcolepsy. In: Goswami M, Pollak CP, Cohen FL, Thorpy MJ, Kavey NB, Kutscher AH, eds. Psychosocial Aspects of Narcolepsy. Vol 5. New York, NY: Haworth; 1992: 23–32.

4.	Flores NM, Villa KF, Black J, Chervin RD, Witt EA. The humanistic and economic burden of narcolepsy. *J Clin Sleep Med.* 2016;**12**(3):401–407. <https://doi.org/10.5664/jcsm.5594>.

5.	Goswami M. The influence of clinical symptoms on quality of life in patients with narcolepsy. *Neurology.* 1998;**50**(2 Suppl 1):S31–36. <https://doi.org/10.1212/wnl.50.2_suppl_1.s31>.

6.	Jones CL. Perceptions of employed people with narcolepsy. <https://scholarworks.waldenu.edu/dissertations>. Accessed 14 July 2025.

7.	Kapella MC, Berger BE, Vern BA, Vispute S, Prasad B, Carley DW. Health-related stigma as a determinant of functioning in young adults with narcolepsy. *PLoS One.* 2015;**10**(4):e0122478. <https://doi.org/10.1371/journal.pone.0122478>.

8.	Maski K, Steinhart E, Williams D*, et al.* Listening to the patient voice in narcolepsy: diagnostic delay, disease burden, and treatment efficacy. *J Clin Sleep Med.* 2017;**13**(3):419–425. <https://doi.org/10.5664/jcsm.6494>.

9.	Rogers AE. Problems and coping strategies identified by narcoleptic patients. *J Neurosurg Nurs.* 1984;**16**(6):326–334. <https://doi.org/10.1097/01376517-198412000-00009>.

10.	Ton TGN, Watson NF, Koepsell TD, Longstreth WT. Narcolepsy and the Sickness Impact Profile: A general health status measure. *Sleep Sci.* 2014;**7**(1):5–12. <https://doi.org/10.1016/j.slsci.2014.07.001>.

11.	Ong JC, Fox RS, Brower RF, Mazurek S, Moore C. How does narcolepsy impact health-related quality of life? A mixed-methods study. *Behav Sleep Med.* 2021;**19**(2):145–158. <https://doi.org/10.1080/15402002.2020.1715411>.

12.	Rodrigues Aguilar AC, Frange C, Huebra L, Dias Gomes AC, Tufik S, Santos Coelho FM. The effects of the COVID-19 pandemic on patients with narcolepsy. *J Clin Sleep Med.* 2021;**17**(4):621–627. <https://doi.org/10.5664/jcsm.8952>.

13.	Rovere H, Rossini S, Reimao R. Quality of life in patients with narcolepsy: a WHOQOL-bref study. *Arq Neuropsiquiatr.* 2008;**66**(2A):163–167. <https://doi.org/10.1590/s0004-282x2008000200004>.

14.	Broughton RJ, Guberman A, Roberts J. Comparison of the psychosocial effects of epilepsy and narcolepsy/cataplexy: a controlled study. *Epilepsia.* 1984;**25**(4):423–433. <https://doi.org/10.1111/j.1528-1157.1984.tb03438.x>.

15.	Jennum P, Knudsen S, Kjellberg J. The economic consequences of narcolepsy. *J Clin Sleep Med.* 2009;**5**(3):240–245.

16.	Jennum P, Ibsen R, Petersen ER, Knudsen S, Kjellberg J. Health, social and economic consequences of hypersomnia: a controlled national study from a national registry evaluating the societal effect on patients and their partners. *Sleep Med.* 2012;**13**(8):1086–1093. <https://doi.org/10.1016/j.sleep.2012.06.006>.

17.	Jennum P, Ibsen R, Kjellberg J. Long-term health and socioeconomic consequences of childhood and adolescent-onset of narcolepsy. *Sleep Med.* 2020;**67**:23–27. <https://doi.org/10.1016/j.sleep.2019.10.013>.

18.	White M, Charbotel B, Fort E*, et al.* Academic and professional paths of narcoleptic patients: the Narcowork study. *Sleep Med.* 2020;**65**:96–104. <https://doi.org/10.1016/j.sleep.2019.07.020>.

19.	Kallweit U, Nilius G, Trümper D, Vogelmann T, Schubert T. Prevalence, incidence, and health care utilization of patients with narcolepsy: a population-representative study. *J Clin Sleep Med.* 2022;**18**(6):1531–1537. <https://doi.org/10.5664/jcsm.9910>.

20.	Müller S, Brandes A, Knierim J*, et al.* Epidemiology, diagnostics, and treatment of narcolepsy in Germany: The DORMIO study. *J Sleep Med.* 2021;**18**(2):88–99. <https://doi.org/10.13078/jsm.210007>.

21.	Dodel R, Peter H, Walbert T*, et al.* The socioeconomic impact of narcolepsy. *Sleep.* 2004;**27**(6):1123–1128. <https://doi.org/10.1093/sleep/27.6.1123>.

22.	Dodel R, Peter H, Spottke A*, et al.* Health-related quality of life in patients with narcolepsy. *Sleep Med.* 2007;**8**(7–8):733–741. <https://doi.org/10.1016/j.sleep.2006.10.010>.

23.	Geremew D, Rahimi-Golkhandan A, Sadeghniiat-Haghighi K*, et al.* Association study of HLA-DQB1*0602 allele in Iranian patients with narcolepsy. *Iran J Allergy Asthma Immunol.* 2017;**16**(5):452–456.

24.	Campbell AJ, Signal TL, O'Keeffe KM, Bakker JP. Narcolepsy in New Zealand: pathway to diagnosis and effect on quality of life. *N Z Med J.* 2011;**124**(1336):51–61.

25.	David A, Constantino F, dos Santos JM, Paiva T. Health-related quality of life in Portuguese patients with narcolepsy. *Sleep Med.* 2012;**13**(3):273–277. <https://doi.org/10.1016/j.sleep.2011.06.021>.

26.	Abenza Abildúa MJ, Suárez Gisbert E, Pérez Villena A, Lores Gutiérrez V, Pérez-López C. Labor impact on patients with narcolepsy. *Sleep Med.* 2023;**103**:98–99. <https://doi.org/10.1016/j.sleep.2023.01.018>.

27.	Daniels E, King MA, Smith IE, Shneerson JM. Health-related quality of life in narcolepsy. *J Sleep Res.* 2001;**10**(1):75–81. <https://doi.org/10.1046/j.1365-2869.2001.00234.x>.

28.	Teixeira VG, Faccenda JF, Douglas NJ. Functional status in patients with narcolepsy. *Sleep Med.* 2004;**5**(5):477–483. <https://doi.org/10.1016/j.sleep.2004.07.001>.

29.	Broughton R, Ghanem Q, Hishikawa Y, Sugita Y, Nevsimalova S, Roth B. Life effects of narcolepsy in 180 patients from North America, Asia and Europe compared to matched controls. *Can J Neurol Sci.* 1981;**8**(4):299–304. <https://doi.org/10.1017/s0317167100043419>.

30.	Broughton R, Ghanem Q, Hishikawa Y, Sugita Y, Nevsimalova S, Roth B. Life effects of narcolepsy: relationships to geographic origin (North American, Asian or European) and to other patient and illness variables. *Can J Neurol Sci.* 1983;**10**(2):100–104. <https://doi.org/10.1017/s0317167100044723>.

31.	Emsellem HA, Thorpy MJ, Lammers GJ*, et al.* Measures of functional outcomes, work productivity, and quality of life from a randomized, phase 3 study of solriamfetol in participants with narcolepsy. *Sleep Med.* 2020;**67**:128–136. <https://doi.org/10.1016/j.sleep.2019.11.1250>.

32.	Thorpy MJ, Ohayon MM, Carls G*, et al.* Assessing the impact of sodium oxybate treatment on functioning, productivity, and health-related quality of life in patients with narcolepsy: findings from the Nexus Narcolepsy Registry (waves 1–4). *Sleep Med.* 2021;**84**:380–388. <https://doi.org/10.1016/j.sleep.2021.06.010>.

33.	Ohayon M, Black J, Gow M*, et al.* P315: Functioning, productivity and health-related quality of life in people with narcolepsy: Results from the ﬁrst eight months of the Nexus Narcolepsy Registry. *J Sleep Res.* 2016;**25**(Suppl 1):190–191. <https://doi.org/10.1111/jsr.12446>.

34.	Weaver TE, Mathias SD, Crosby RD*, et al.* Relationship between sleep efficacy endpoints and measures of functional status and health-related quality of life in participants with narcolepsy or obstructive sleep apnea treated for excessive daytime sleepiness. *J Sleep Res.* 2021;**30**(3):e13210. <https://doi.org/10.1111/jsr.13210>.

35.	Weaver TE, Pepin J-L, Schwab R*, et al.* Long-term effects of solriamfetol on quality of life and work productivity in participants with excessive daytime sleepiness associated with narcolepsy or obstructive sleep apnea. *J Clin Sleep Med.* 2021;**17**(10):1995–2007. <https://doi.org/10.5664/jcsm.9384>.

36.	Alaia SL. Life effects of narcolepsy: measures of negative impact, social support, and psychological well-being. In: Psychosocial Aspects of Narcolepsy. Vol 5. Routledge; 1992: 1–22.

37.	Kales A, Soldatos CR, Bixler EO*, et al.* Narcolepsy-cataplexy. II. Psychosocial consequences and associated psychopathology. *Arch Neurol.* 1982;**39**(3):169–171. <https://doi.org/10.1001/archneur.1982.00510150039009>.

38.	Kovalská P, Kemlink D, Nevšímalová S*, et al.* Narcolepsy with cataplexy in patients aged over 60 years: a case-control study. *Sleep Med.* 2016;**26**:79–84. <https://doi.org/10.1016/j.sleep.2016.05.011>.

39.	Peter-Derex L, Fort E, Putois B*, et al.* Academic and socio-professional trajectories in narcolepsy type 1: the NARCOSCOL-NARCOVITAE multicentric study. *medRxiv.* 2024. <https://doi.org/10.1101/2024.01.13.24301265>.

40.	Peter-Derex L, Fort E, Putois B*, et al.* Effort/reward imbalance and comorbidities burden in academic and professional careers of patients with narcolepsy type 1. *J Clin Sleep Med.* 2025;**21**(6):983-997. <https://doi.org/10.5664/jcsm.11598>.

41.	Ingravallo F, Gnucci V, Pizza F*, et al.* The burden of narcolepsy with cataplexy: How disease history and clinical features influence socio-economic outcomes. *Sleep Med.* 2012;**13**(10):1293–1300. <https://doi.org/10.1016/j.sleep.2012.08.002>.

42.	Ingravallo F, Vignatelli L, Brini M*, et al.* Medico-legal assessment of disability in narcolepsy: an interobserver reliability study. *J Sleep Res.* 2008;**17**(1):111–119. <https://doi.org/10.1111/j.1365-2869.2008.00630.x>.

43.	Bassi C, Biscarini F, Zenesini C*, et al.* Work productivity and activity impairment in patients with narcolepsy type 1. *J Sleep Res.* 2024;**33**(3):e14087. <https://doi.org/10.1111/jsr.14087>.

44.	Overeem S, van Nues SJ, van der Zande WL, Donjacour CE, van Mierlo P, Lammers GJ. The clinical features of cataplexy: A questionnaire study in narcolepsy patients with and without hypocretin-1 deficiency. *Sleep Med.* 2011;**12**(1):12–18. <https://doi.org/10.1016/j.sleep.2010.05.010>.

45.	Ervik S, Abdelnoor M, Heier MS, Ramberg M, Strand G. Health-related quality of life in narcolepsy. *Acta Neurol Scand.* 2006;**114**(3):198–204. <https://doi.org/10.1111/j.1600-0404.2006.00594.x>.

46.	Stevens J, Schneider LD, Husain AM*, et al.* Impairment in functioning and quality of life in patients with idiopathic hypersomnia: The Real World Idiopathic Hypersomnia Outcomes Study (ARISE). *Nat Sci Sleep.* 2023;**15**:593–606. <https://doi.org/10.2147/NSS.S396641>.

47.	Broughton R, Nevsimalova S, Roth B. The socio-economic effects of idiopathic hypersomnia – comparison with controls and with compound narcoleptics. *Sleep.* 1978:229–233.

48.	Jennum P, Kjellberg J. The socio-economical burden of hypersomnia. *Acta Neurol Scand.* 2010;**121**(4):265–270. <https://doi.org/10.1111/j.1600-0404.2009.01227.x>.

49.	Jennum P, Ibsen R, Avlund K, Kjellberg J. Health, social and economic consequences of hypersomnia: a controlled national study from a national registry evaluating the societal effect on patients and their partners. *Eur J Health Econ.* 2014;**15**(3):303–311. <https://doi.org/10.1007/s10198-013-0491-2>.

50.	Morse AM, Dauvilliers Y, Arnulf I*, et al.* Long-term efficacy and safety of low-sodium oxybate in an open-label extension period of a placebo-controlled, double-blind, randomized withdrawal study in adults with idiopathic hypersomnia. *J Clin Sleep Med.* 2023;**19**(10):1811–1822. <https://doi.org/10.5664/jcsm.10698>.

51.	Thorpy MJ, Arnulf I, Foldvary-Schaefer N*, et al.* Efficacy and safety of lower-sodium oxybate in an open-label titration period of a phase 3 clinical study in adults with idiopathic hypersomnia. *Nat Sci Sleep.* 2022;**14**:1901–1917. <https://doi.org/10.2147/NSS.S369122>.

52.	Srivastava B, Morris S, Banderas B, Lowe C, Dauvilliers Y. Patient-reported symptoms and health-related quality of life impacts of idiopathic hypersomnia. *Ann Neurol.* 2021;**90**(Suppl 27):S240–S241. <https://doi.org/10.1002/ana.26180>.

53.	Susta M, Šonka K, Bizik G, Petranek S, Nevsimalova S. Idiopathic hypersomnia-A dynamic simulation model. *Front Neurol.* 2022;**13**:902637. <https://doi.org/10.3389/fneur.2022.902637>.

54.	Ozaki A, Inoue Y, Hayashida K*, et al.* Quality of life in patients with narcolepsy with cataplexy, narcolepsy without cataplexy, and idiopathic hypersomnia without long sleep time: Comparison between patients on psychostimulants, drug-naïve patients and the general Japanese population. *Sleep Med.* 2012;**13**(2):200–206. <https://doi.org/10.1016/j.sleep.2011.07.014>.

55.	Wasling HB, Bornstein A, Wasling P. Quality of life and procrastination in post-H1N1 narcolepsy, sporadic narcolepsy and idiopathic hypersomnia, a Swedish cross-sectional study. *Sleep Med.* 2020;**76**:104–112. <https://doi.org/10.1016/j.sleep.2020.10.014>.

1. A total of 73 narcoleptic patients were identified from the departmental database that met the age criterion and had a diagnosis of NT1. Of these, 29 regularly attended the clinic, ≥1 per year, and 5 attended irregularly; all were included in the study. Of the remaining patients, 18 were successfully contacted who had not attended the department for years (8 were included in the study, 10 refused to participate due to difficulties in reaching the hospital because of generally poor health or mobility, and/or residence far from the department, and/or low motivation), and 6 were found to be deceased.
